# Ion–Electron Coupling Enables Ionic Thermoelectric Material with New Operation Mode and High Energy Density

**DOI:** 10.1007/s40820-023-01077-7

**Published:** 2023-04-13

**Authors:** Yongjie He, Shaowei Li, Rui Chen, Xu Liu, George Omololu Odunmbaku, Wei Fang, Xiaoxue Lin, Zeping Ou, Qianzhi Gou, Jiacheng Wang, Nabonswende Aida Nadege Ouedraogo, Jing Li, Meng Li, Chen Li, Yujie Zheng, Shanshan Chen, Yongli Zhou, Kuan Sun

**Affiliations:** MOE Key Laboratory of Low-grade Energy Utilization Technologies and Systems, CQU-NUS Renewable Energy Materials and Devices Joint Laboratory, School of Energy and Power Engineering, Chongqing University, Chongqing, 400044 People’s Republic of China

**Keywords:** Ionic thermoelectric, Ion–electron coupling, Ionic conductivity, Thermopower

## Abstract

Ionic thermoelectrics (i-TE) possesses great potential in powering distributed electronics because it can generate thermopower up to tens of millivolts per Kelvin. However, as ions cannot enter external circuit, the utilization of i-TE is currently based on capacitive charge/discharge, which results in discontinuous working mode and low energy density. Here, we introduce an ion–electron thermoelectric synergistic (IETS) effect by utilizing an ion–electron conductor. Electrons/holes can drift under the electric field generated by thermodiffusion of ions, thus converting the ionic current into electrical current that can pass through the external circuit. Due to the IETS effect, i-TE is able to operate continuously for over 3000 min. Moreover, our i-TE exhibits a thermopower of 32.7 mV K^−1^ and an energy density of 553.9 J m^−2^, which is more than 6.9 times of the highest reported value. Consequently, direct powering of electronics is achieved with i-TE. This work provides a novel strategy for the design of high-performance i-TE materials.

**Supplementary Information:**

The online version contains supplementary material available at 10.1007/s40820-023-01077-7.

## Highlights

- An ion–electron coupled thermoelectric material was successfully prepared, which theoretically proved the ion–electron thermoelectric synergy effect and this material can work for a long time, which promoted low-grade thermal energy conversion.
- In the new operating mode of ion–electron thermoelectric synergy effect, our ionic thermoelectrics have a high Seebeck coefficient of 32.7 mV K^−1^ and a high energy density of 553.9 J m^−2^, enabling self-power for electronic components.

**Supplementary Information:**

The online version contains supplementary material available at 10.1007/s40820-023-01077-7.

## Graphical abstract

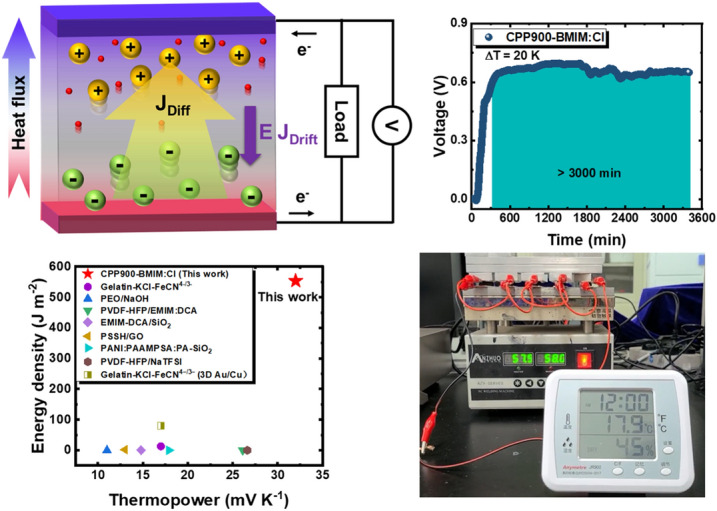

## Introduction

Harvesting energy from environment to power distributed electronics is long desired for Internet-of-Things [1–3]. Through Seebeck effect, which refers to the establishment of electrical potential difference under a temperature gradient, one can realize direct conversion of heat to electricity without any moving part or emission [4, 5]. To this end, electronic thermoelectrics (e-TE) [6, 7], thermocells (TC) [8–10] and ionic thermoelectrics (i-TE) [11, 12] have shown great potential in such an application. High-performance thermoelectric materials possess both high thermopower (also known as Seebeck coefficient) (*S*) and high electrical conductivity (*σ*), but low thermal conductivity (*κ*) [13].i-TEs have attracted extensive attention due to their ability to generate thermopower of tens of millivolts per Kelvin [11, 14–28], ideal for thermal energy recovery near room temperature. The working principle of i-TEs is based on Soret effect, i.e., ions in the electrolyte thermo-diffuse from the hot side to the cold side driven by a temperature gradient, thus inducing an ionic concentration gradient and an electric field. Since ions cannot enter external circuit, the application of i-TE generally relies on the capacitance formed by the ions accumulated at the electrodes for thermal charging and discharging [12]. However, this working mechanism results in discontinuous operation [11, 12, 18, 23] and a low power output [29], hindering its direct application.

There are two strategies that have been explored to enhance the power output of i-TEs, i.e., increasing the specific surface area of the electrode [12] or coupling ions and electrons [30, 31]. The highest energy density adopted the new strategies reached 80 J m^−2^ [32]. Nevertheless, the energy density is still low compared with e-TEs. Moreover, the working mode is still based on discontinuous thermal charging/discharging. Thus, it is vital to seek a new operation mechanism that can boost thermoelectric performance of i-TEs.

In this work, we tackle the problem by introducing a new operation mechanism based on an ion–electron thermoelectric synergistic (IETS) effect. This new mechanism relies on the utilization of an ion–electron conductor. Under a thermal gradient, thermodiffusion of ions in i-TE will set up an electric potential; since the scaffold is electrically conducting, electrons/holes in the scaffold can also drift under the electric field generated by the ions. Through this IETS effect, one can convert the ionic current into electrical current that can pass through the external circuit to do work. Due to the IETS effect, i-TE is able to operate continuously for over 3000 min without the aid of an energy storage device. Moreover, our i-TE exhibits a thermopower of 32.7 mV K^−1^ and an energy density of 553.9 J m^−2^, which is more than 6.9 times of the highest reported value. Consequently, direct powering of electronics is demonstrated with IETS-based i-TE. This work provides a novel strategy for the design of high-performance i-TE materials.

## Experimental Section

### Materials

BMIM:Cl (*M*_w_ = 174.671 g mol^−1^, > 99.9%) was purchased from Adamas Reagent. Ethanol (*M*_w_ = 46.07 g mol^−1^, > 99.7%) was purchased from Chengdu Kelong Chemical Co., Ltd. The pomelo peels used in this article were purchased from Jiangxi, China. All materials were used without further purification.

### Preparation of CPP-BMIM:Cl

Fresh grapefruit peels were removed from the outer epidermis, washed with deionized water, frozen, and vacuum dried for 48 h. Then treated under nitrogen atmosphere at 900/500 °C for 2 h to obtain CPP900/500. The pomelo peel was heated at a rate of 10 °C min^−1^. Afterward, CPP900/500 samples were soaked in ethanol for 1 h and then cut into specific size (~ 5 mm × 9 mm × 14 mm). Finally, the CPP900/500 was immersed in 1 M BMIM:Cl aqueous solution and left for 24 h to complete the preparation of CPP-BMIM:Cl.

### Performance Characterization of CPP900, CPP900-BMIM:Cl and CPP500-BMIM:Cl (Hereinafter Referred to as CPPs)

The thermopower was derived by dividing the obtained thermally generated open circuit voltage by the temperature difference across the device. CPPs were sandwiched between two flat copper electrodes, the bottom side was heated and the top side was passively cooled in air. Two thermocouples were adhered inside the CPPs and in contact with the electrodes. The temperature difference was recorded using UNI-T 325. A Keithley 2400 was used to measure the open circuit voltage or short circuit current between the two electrodes. The resistance (*R*) of CPP900 was tested by Keithley 2400, and the electrical conductivity ($\sigma$) of CPP900 was obtained via the following formula:

1 $$\begin{array}{*{20}c} {\sigma = \frac{1}{R}\frac{l}{A}} \\ \end{array}$$

where $\sigma$ is the electrical conductivity, *R* is the resistance, $l$ is the thickness of CPP and *A* is the area. The ionic conductivity of the CPPs was determined by the electrochemical impedence spectroscopy (EIS). Using the same setup to perform potentiostatic scanning with a voltage amplitude of 10 mV, and a frequency ranging from 0.1 Hz to 100 kHz.

### Material Characterization

For cyclic voltammetry (CV) scan, the CPPs were sandwiched between two flat copper electrodes, and the voltage window from − 0.8 to + 0.8 V was measured by Bio-logic (Gamry biologic, America), in which one platinum sheet served as the working electrode while the other one served as the counter and reference electrodes simultaneously. The scan rate was 20, 40, 60, 80 and 100 mV s^−1^. The morphologies and microstructures of CPPs were characterized by SEM (TM4000Plus II, Hitachi LTD). FTIR transmittance spectroscopy test was employed with a FTIR spectrometer (Spectrum Two, PerkinElmer) in the solid test mode. XRD were tested by PANalytical X’Pert Powder. XPS were performed with an ESCALAB-250Xi X-ray photoelectron spectrometer. BET was performed with Quadrasorb 2MP.

## Results and Discussion

**Fig. 1:**
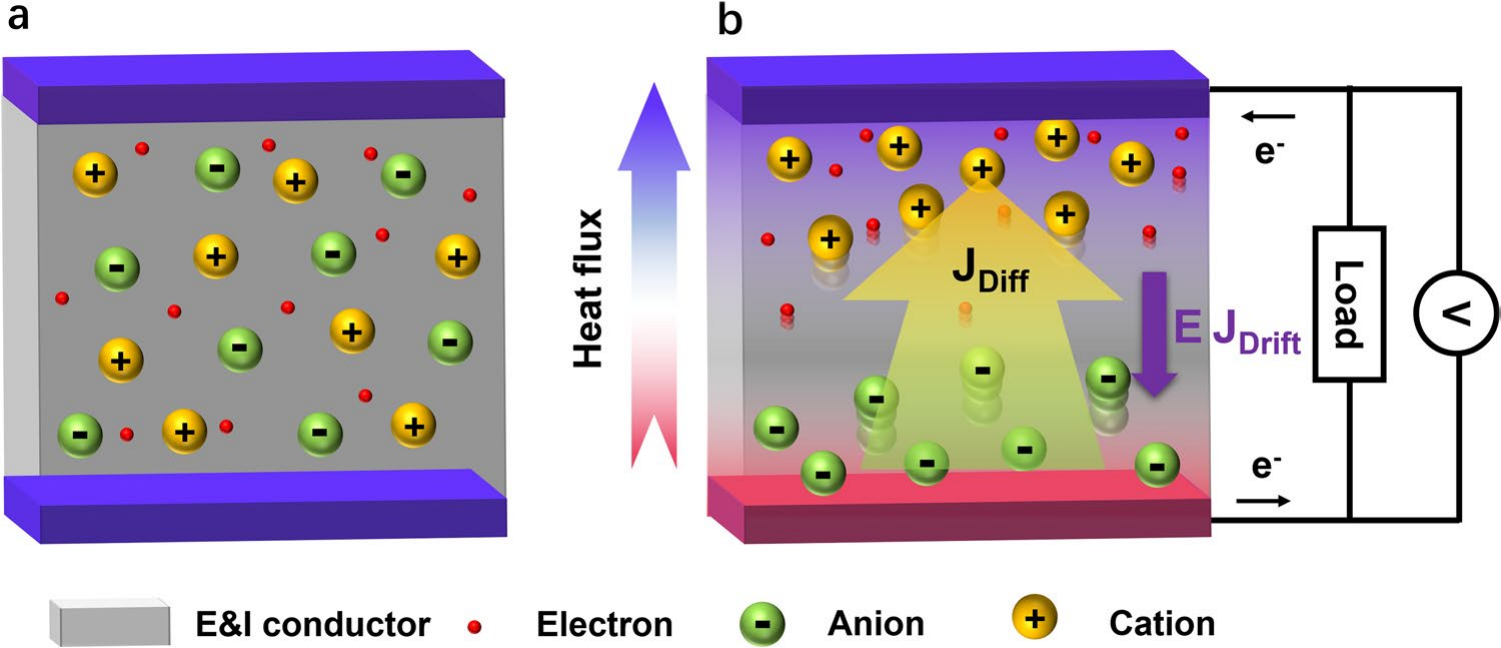
**a** Schematic diagram of the ion–electron conductor without an applied temperature gradient, the ions and electrons are evenly distributed, and there is no potential difference. The electrons here refer to the carrier in the E&I conductor. **b** Schematics of the working principle under the condition of ion–electron thermoelectric synergy

In this study, we couple Soret effect and drift current to exploit a new i-TE operation principle (Supporting Information and Fig. S1). We introduced a dual functional scaffold made from carbonized pomelo peel (CPP), which could conduct both ions and electrons. Such a scaffold was regarded as an ion–electron conductor (IE conductor). Our i-TE material was prepared by filling an ionic liquid, 1-butyl-3-methylimidazole chloride (BMIM:Cl), into the porous CPP (Fig. S2). Figure 1a depicts the i-TE without an applied temperature gradient. The ions and electrons are homogeneously distributed in the system, so there is no electrical potential generated. Once a temperature difference is applied to this i-TE (Fig. 1b), the thermo-diffusion of ions induces an ionic diffusion current and an electric field due to Soret effect. Driven by this electric field, a drift current is generated inside the IE conductor, whose flow direction is the same as the electric field [3]. It is worth mentioning that the driving force for the ionic current is the temperature gradient, while the driving force for the drift current is the electric field generated by the ions. In this way, when the i-TE is connected to an external circuit, part of the drift current goes through the external load, hereby converting the ionic current to electrical current that can flow into external circuit. We regard this working mode as ion–electron thermoelectric synergy (IETS) effect. We further derived the total expression of the thermopower under the IETS effect as follows (full derivations are described in Supporting Information):

2 $$\begin{array}{*{20}c} {S = \frac{\Delta V}{{\Delta T}} = \frac{q}{{\sigma_{{\text{e - TE}}} }}\left( {D_{ - } C_{ - } S_{T - } - D_{ + } C_{ + } S_{T + } } \right)} \\ \end{array}$$

where *q* is the elemental charge,* σ*_e-TE_ is the electrical conductivity of the IE conductor, *D* is the diffusion coefficient of the ion, *C* and *S*_*T*_ are the ion concentration and the Soret coefficient, respectively. The subscript “ + ” or “ − ” denotes the ion species.

From Eq. (2), we note that given a specific temperature gradient:

2a $$\begin{array}{*{20}c} {\Delta V \propto \frac{1}{{\sigma_{{\text{e - TE}}} }}} \\ \end{array}$$

2b $$\begin{array}{*{20}c} {\Delta V \propto \left( {D_{ - } C_{ - } S_{T - } - D_{ + } C_{ + } S_{T + } } \right)} \\ \end{array}$$

We also note that the expression for the drift current is:

3 $$\begin{array}{*{20}c} {J_{{{\text{Drift}}}} = \sigma_{{\text{e - TE}}} \cdot E = \sigma_{{\text{e - TE}}} \frac{{{\text{d}}V}}{{{\text{d}}x}}} \\ \end{array}$$

Based on Eqs. (2a) and (3), one can deduce that the electrical conductivity of the IE conductor cannot be too large nor too small under the IETS working principle. A large conductivity can quickly balance out the voltage generated by ions [31], reducing the thermovoltage; while a small conductivity will result in low current output. As implied by Eq. (2b), the Soret effect still plays a role in IETS working mode, i.e., increasing the ion’s Eastman entropy of transfer increases the ionic thermopower.

To prove the IETS concept, we fabricated i-TE materials incorporated with an ion conductor or an IE conductor, for a comparison. The different charge conductors were obtained by carbonizing fresh pomelo peels at different temperatures, e.g., 500 and 900 °C, denoted as CPP500 and CPP900. CPP900 exhibits an electrical conductivity of 31.2 S m^−1^ (Fig. S3), while CPP500 is an electrical insulator as the measured resistance is overloaded. This is because an increase in carbonization temperature enhances the degree of graphitization of pomelo peels [33]. Besides, the porous CPP contains a large number of hydroxyl or carbonyl groups, which render it a good ion conductor [34]. So CPP500 is an ion conductor, while CPP900 is an IE conductor. The i-TE material was made by filling CPP with an aqueous solution of BMIM:Cl, denoted as CPP-BMIM:Cl.

**Fig. 2:**
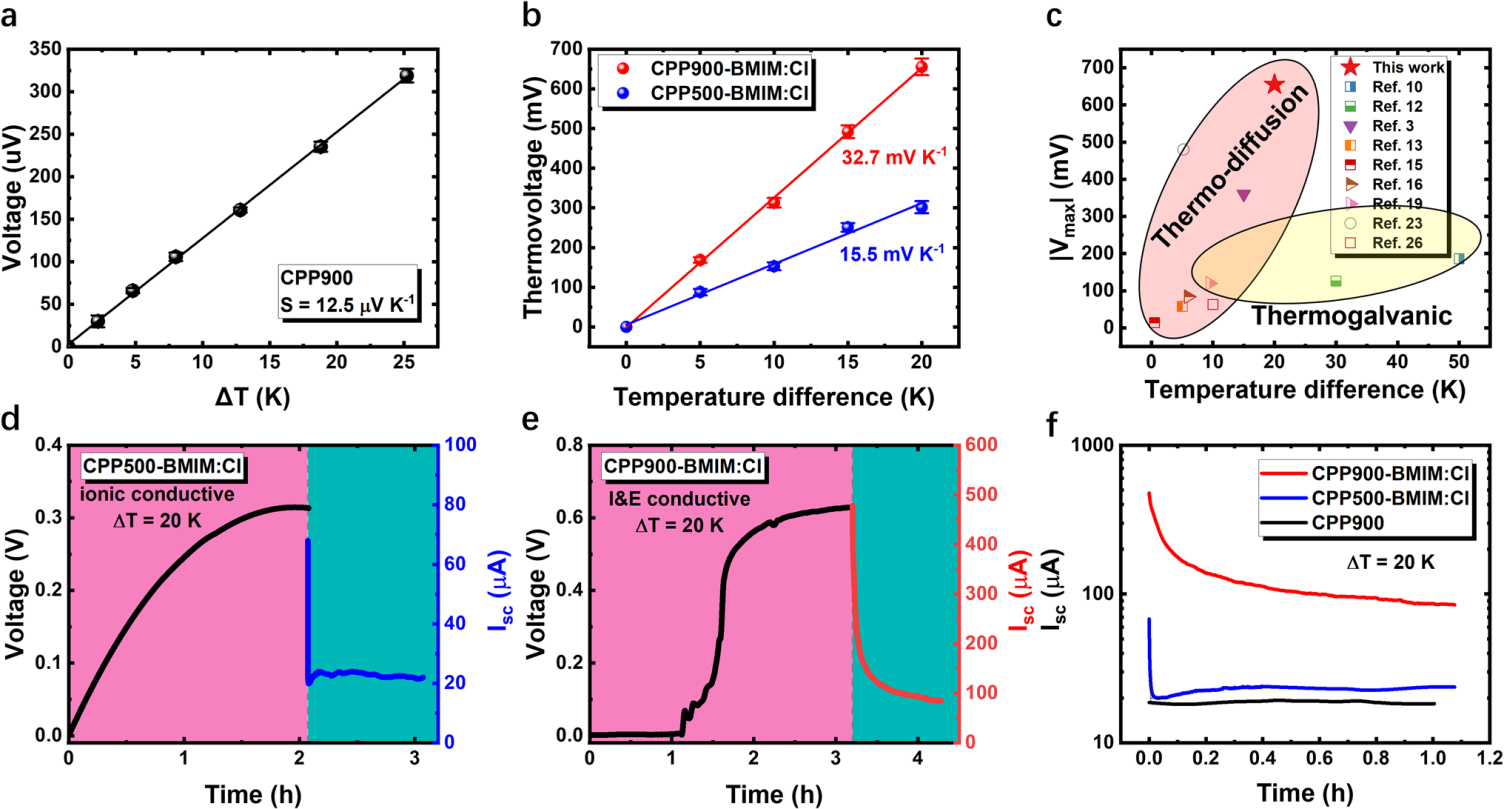
**a** The thermopower of CPP900. **b** The thermopower of CPP500-BMIM:Cl and CPP900-BMIM:Cl determined by a liner fit of the open circuit voltage verses temperature difference. **c** Absolute thermovoltage of a single i-TE compared to reported values. The thermovoltage resulted from ion thermodiffusion effect is shaded in pink, and the thermovoltage due to thermogalvanic effect is shaded in yellow. Open circuit thermovoltage curve and short circuit current curve of **d** CPP500-BMIM:Cl and **e** CPP900-BMIM:Cl. The cell size is 9 mm × 14 mm. **f** A comparison of short-circuit current among CPP900, CPP500-BMIM:Cl and CPP900-BMIM:Cl after thermal charging. (Color figure online)

CPP900 exhibits a low thermopower of 12.5 μV K^−1^ (Fig. 2a) and an instantaneous thermovoltage response (Fig. S4), so CPP900 alone can be regarded as an e-TE material [35]. In a great contrast, the thermopower of CPP900-BMIM:Cl is 32.7 mV K^−1^ and that of CPP500-BMIM:Cl is 15.5 mV K^−1^ (Figs. 2b and S5, S6). The large thermopower indicates the CPP-BMIM:Cl composite is predominantly an i-TE material [1]. It is worth noting that the thermovoltage of a single CPP900-BMIM:Cl cell reaches 0.65 V under a temperature difference of 20 K (Fig. 2b). To the best of our knowledge, this is currently the highest thermovoltage achieved by a single i-TE (Fig. 2c; Table S1).

A series of comparative experiments were carried out to confirm that the thermopower was induced by ionic thermo-diffusion. The thermopower of the CPP900-BMIM:Cl is orientation-independent, i.e., the CPP900-BMIM:Cl achieves the same thermopower value when the hot and cold electrode are interchanged (Fig. S7). When only deionized water was introduced into CPP900, a low thermovoltage of 478 μV is observed under the same temperature difference of 20 K (Fig. S8), indicating the ions in this system play a key role for the large thermopower [36, 37]. The thermopower of BMIM:Cl neat solution without CPP scaffold is just 0.35 ± 0.05 mV K^−1^, which highlights the importance of CPP in ionic thermopower, and implies the possible interaction between solution and electrode has little effect on ionic thermopower (Fig. S9). In addition, the saturation of thermovoltage in neat BMIM:Cl solution is significantly faster than that in CPP-BMIM:Cl, indicating that the selective effect of CPP on ions leads to a longer time to reach voltage equilibrium. Cyclic voltammetry measurement was performed to further show that no redox reaction takes place (Fig. S10) and the valence state of the ions remains unchanged [11].

Subsequently we studied the thermovoltage response (charging) of CPP500-BMIM:Cl and CPP900-BMIM:Cl. The thermovoltage of CPP500-BMIM:Cl reaches 0.31 V (Fig. 2d) within 2 h and CPP900-BMIM:Cl reaches 0.64 V within 3 h (Fig. 2e). In addition, we notice an initial time-lag in the thermovoltage build-up for CPP900-BMIM:Cl, while this phenomenon is absent in the thermovoltage response of CPP500-BMIM:Cl. Repetitive measurements (Fig. S11), confirm that the initial time-lag is a characteristic of all CPP900-BMIM:Cl samples. This unique characteristic is attributed to the decrease in electrical conductivity during the process of ionic thermal diffusion (Fig. S12), which in turn weakens the ability of electrons to balance the ionic potential difference [38].

We further compared the current output characteristics (discharge) of CPP500-BMIM:Cl, CPP900-BMIM:Cl and CPP900 (Fig. 2f). After thermal charging at a temperature difference of 20 K, the short-circuit current of CPP500-BMIM:Cl decays from 68 to 20 ${\mu A}$ (point of inflection) within 15 s; while that of CPP900-BMIM:Cl gradually decays from 478 to 124 ${\mu A}$ (point of inflection) in 195 s. For a comparison, the short-circuit current of CPP900 is almost constant at 19 ${\mu A}$ due to its e-TE nature. The fast decay of current output implies that CPP500-BMIM:Cl operates in an inductive capacitance mode, i.e., the accumulated ions at the hot and cold electrodes induce current flow in the external circuit [24]. The boost in current output of CPP900-BMIM:Cl is mainly attributed to the IETS effect. When the scaffold of CPP900-BIMI:Cl is conductive, part of the drift current can go through the external circuit, greatly contributing to the current output. Since CPP900 can only generate a constant but small current, we can rule out the possibility that the large current output of CPP900-BMIM:Cl is due to the contribution from the intrinsic thermoelectric current of CPP900. The above discussion indicates that the IETS effect can greatly improve the current output.

It is worth mentioning that a measurable short-circuit current can be observed instantly for all three samples as soon as the temperature gradient is applied (Fig. S13). Under a temperature difference of 20 K, CPP900 delivers a constant current of 19 ${\mu A}$. Both CPP500-BMIM:Cl and CPP900-BMIM:Cl show an increasing output current, with the latter increases to more than 450 ${\mu A}$ after one hour. The small current increase of CPP500-BMIM:Cl is attributed to the slow ion accumulation at the electrode that generates a capacitor-like current. Meanwhile, the immediate current increase of CPP900-BMIM:Cl indicates that upon the applied temperature difference, an induced sizable drift current is generated instantly. We can clearly recognize that when BMIM:Cl is introduced, CPP has a significant improvement in both thermovoltage output and current output.

**Fig. 3:**
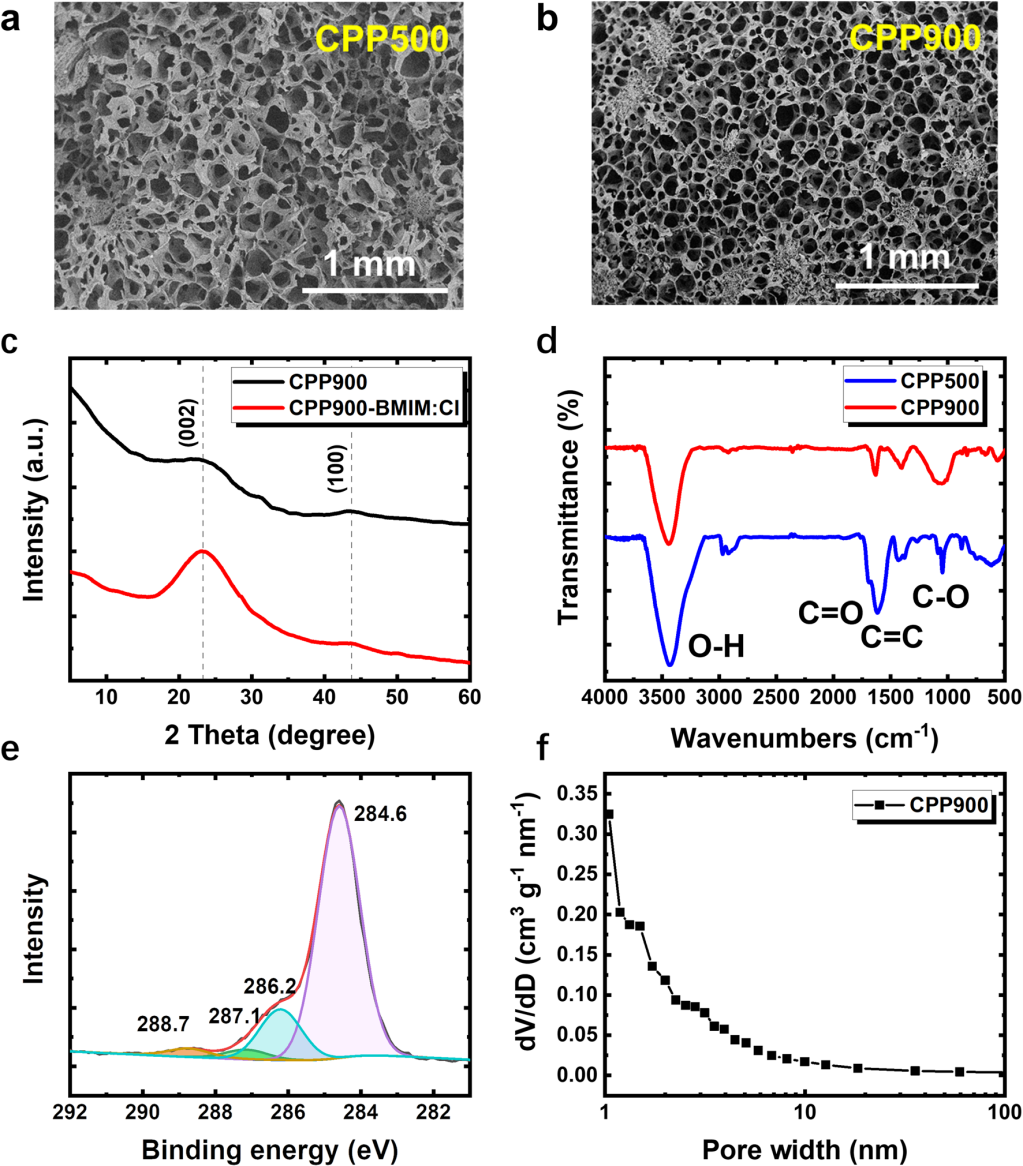
**a**, **b** SEM image of CPP500 and CPP900. The scale bar is 1 mm.** c** XRD patterns of the CPP900 and CPP900-BMIM:Cl. **d** FTIR spectra of CPP900 and CPP500. **e** The C 1*s* XPS spectra of CPP900-BMIM:Cl. **f** Pore size distribution of CPP900

To understand the origin of the giant thermopower and the difference in thermoelectric performance between CPP900-BMIM:Cl and CPP500-BMIM:Cl, structure in multi-scales of CPPs were characterized. Scanning electron microscopic (SEM) images in Fig. 3a, b demonstrate that the graphitized pomelo peel exhibits a porous structure at micron scale. The microstructure of CPP exhibits isotropic polydispersity in pore-size and nanofiber thickness (Fig. S14a), implying an isotropic thermoelectric property, in contrast to some anisotropic biomass derivatives such as wood [39] and bamboo [40]. CPP900 possesses finer nanofibers and denser pore distribution compared to CPP500 (Figs. 3a, b and S14b, c). This may be resulted from the differentiation of nanofibers at higher temperatures. The XRD pattern of CPP exhibits two broadened peaks at 22.9° and 43.7° (Fig. 3c), corresponding to the {002} and {100} planes of graphite [33], respectively. It shows that CPP is an amorphous graphite-like structure [41].

The results of Fourier transform infrared spectroscopy (FTIR) indicate that CPP500 and CPP900 have similar functional groups (Fig. 3d). The FTIR bands show that O–H, C=O, C=C and C–O bonds are present in CPP, implying the existence of various functional groups, such as –OH, –COOH, etc. These polar functional groups endow the CPP with the ability to conduct ions [42]. When BMIM:Cl was introduced, the O–H peak position is red shifted (Fig. S15), indicating that BMIM^+^ formed hydrogen bonds with –OH [43]. X-ray photoelectron spectroscopy (XPS) further confirms the existence of oxygen-containing functional groups (Figs. 3e and S16). The C 1*s* spectrum could be deconvoluted into 4 peaks centered at 284.6, 286.2, 287.1 and 288.7 eV, which were assigned to carbon in aromatic or aliphatic structures; phenol, ether, or enol–keto groups; carbonyl or quinone; and carboxyl or ester groups, respectively [34].

In addition, we compared the pore size distribution of CPP500 and CPP900. Brunauer Emmett Teller (BET) test results show that CPP900 has on average smaller micro–nano-structured pores and larger specific surface area (485.04 m^2^ g^−1^) than CPP500 (0.65 m^2^ g^−1^) (Figs. 3f and S17), which provide more pathways and connectivity for ion transport. This may account for the difference in their thermopower performance. In addition, the large difference in the specific surface area of CPP900 and CPP500 is also one of the reasons for the difference in discharge behavior.

Based on the above results, we provide an insight into the colossal thermopower induced by the ion diffusion of the CPP-BMIM:Cl. The presence of oxygen-containing functional groups in CPP makes the surface negatively charged (Fig. S18). The ionic interaction induced by the charged CPP surface can generate a large Eastman entropy of transfer, which is responsible for the large thermopower [18]. In addition, both experiments [44] and computational analysis [45] have shown that oxygen-containing functional groups are more favorable for cation diffusion, and the greater the negative charge density, the stronger the ion selectivity [11, 46]. The thermodiffusion of BMIM:Cl induces an accumulation of BMIM^+^ at the cold electrode due to the selectivity of CPP, resulting in a *p*-type thermopower.

**Fig. 4:**
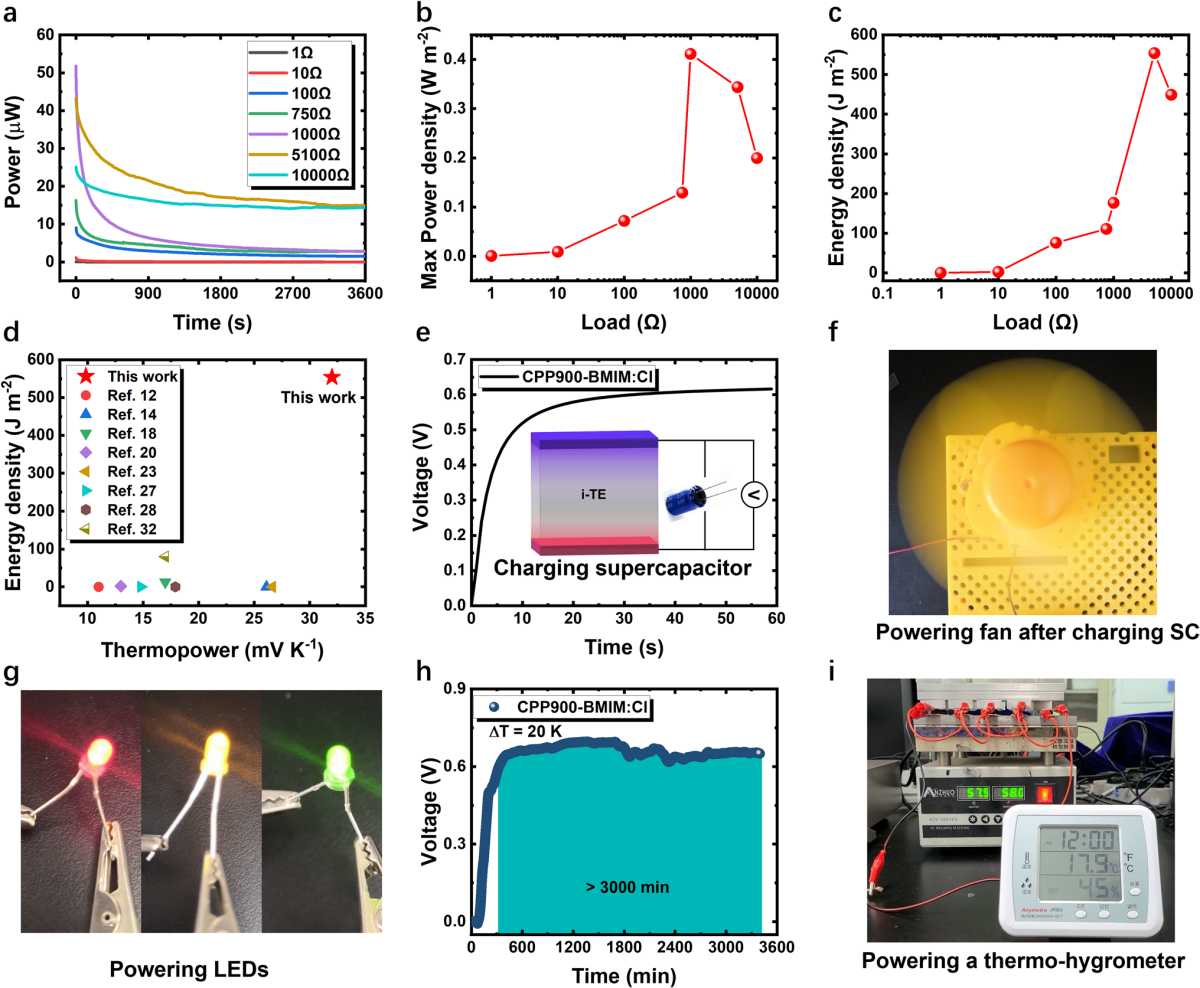
**a** Power curves of CPP900-BMIM:Cl during discharging when connected to different external loads. The temperature difference is 20 K. Variation trend of **b** maximum output power and **c** energy density with external load. **d** Comparison of energy density of CPP900-BMIM:Cl with the optimal values reported in literatures. Because the test conditions for i-TE energy density are not uniform (discharge time, temperature difference, load resistance, relative humidity, etc.), we provide a rough comparison here to facilitate an understanding of the output limit and scale of i-TEs. **e** Charging curve of CPP900-BMIM:Cl for a 10 mF supercapacitor at a temperature difference of 20 K. **f** A fully charged supercapacitor with a capacity of 0.5 F can drive a small fan directly. **g** Connecting 4 charged supercapacitors (10 mF) in series, the recovered heat can light up red, yellow and green light-emitting diodes. **h** The open-circuit voltage of CPP900-BMIM:Cl under a continuous temperature difference of 20 K. **i** Powering a thermo-hygrometer directly by a i-TE module without supercapacitors. (Color figure online)

Since CPP900-BMIM:Cl delivers both large thermovoltage and short-circuit current, its power output behavior was then evaluated. Figure S19 displays the discharge voltage curves of different external loads after the CPP900-BMIM:Cl was thermally charged at 20 K. The voltage slowly decays to a nonzero constant value in all these scenarios, unlike ionic thermoelectric capacitors that gradually decay to zero [17, 26]. This indicates that the i-TE working in IETS mode can generate a stable output. In contrast, without the introduction of BMIM:Cl, the voltage output of the CPP900 stays at the microvolt level (Fig. S20). This highlights the importance of IETS effect. The output power profiles of CPP900-BMIM:Cl was obtained via Ohm’s law, as shown in Fig. 4a. When the external load is 1000 ohms, the maximum power density of CPP900-BMIM:Cl reaches 0.41 W m^−2^ (Fig. 4b). To the best of our knowledge, this is the highest power density for i-TE reported so far (Fig. S21; Table S2). The discharged energy of CPP900-BMIM:Cl in one hour was calculated by integrating the power versus time curve. Under an external load of 5100 ohms, the energy density is as high as 553.9 J m^−2^ (Fig. 4c), which is more than 6.9 times the best reported i-TE (Fig. 4d and Table S2).

To further verify the credibility of the power output of CPP900-BMIM:Cl, it was connected with a supercapacitor. The voltage of the supercapacitor increased to the output voltage of the CPP900-BMIM:Cl within 1 min (Fig. 4e), indicating that the CPP900-BMIM:Cl could easily charge a supercapacitor. After charging a 0.5 F supercapacitor (Fig. S22), the waste heat collected by CPP900-BMIM:Cl can drive a fan directly (Fig. 4f). Collecting 4 supercapacitors in series, the output voltage is high enough to power light-emitting diodes (Fig. 4g).

Due to the IETS effect, CPP900-BMIM:Cl can convert heat into electricity, and output both voltage and current continuously for more than 3000 min (Figs. 4h and S23), showing good working durability. After continuous power generation, it could be easily restored (Fig. S24) and remain operational for consecutive days (Fig. S25). This demonstrates that our i-TE material can be reused, not a one-time energy source. These results demonstrate that the IETS effect provides i-TEs with a completely new operation mode, which is fundamentally different from traditional i-TE materials. The traditional i-TE materials require four-stage switch of heat and circuit [12, 19, 32, 47]; while the i-TEs with IETS effect can work continuously for over 50 h once the thermal gradient is established.

Finally, we designed an i-TE module by connecting 5 i-TE cells (size: 5 mm × 9 mm × 14 mm) in series (Fig. S26). Because of the considerable power output of CPP900-BMIM:Cl, this module can directly power a thermo-hygrometer without using supercapacitors under a temperature difference of 20 K. (Fig. 4i). The device can also achieve continuous output. In Supplementary Video, we demonstrate the i-TE module is able to drive a thermo-hygrometer incessantly for 1 h. This demonstration clearly shows IETS effect can bring about a brand-new operation mode for i-TE materials, which is ideal for IoT devices.

## Conclusions

In summary, unlike previously reported i-TE energy conversion approaches, we enhanced the power output of i-TE by introducing an ion–electron (IE) conductor to promote the ion–electron thermoelectric synergy (IETS) effect. We further derived the working mechanism of the IETS effect, providing guidance for new i-TE materials design. As a proof of concept, CPP900-BMIM:Cl that utilizes IETS effect achieved a high thermopower of 32.7 mV K^−1^, a record thermovoltage of 0.65 V, a record power density of 0.41 W m^−2^ and a record energy density of 553.9 J m^−2^. The i-TE material can also operate continuously for over 3000 min and can be easily restored and reused. Eventually, we fundamentally changed the working mode of traditional i-TE modules, demonstrating direct powering of electronics by an i-TE module in a continuous and stable fashion, without using supercapacitors.

### Supplementary Information

Below is the link to the electronic supplementary material.

Supplementary file1 (PDF 2270 KB)

Supplementary file2 (PDF 2923 KB)
