# Supplementary material for: Ion–Electron Coupling Enables Ionic Thermoelectric Material with New Operation Mode and High Energy Density
Source: Nanomicro Lett. 2023 Apr 13;15:101. doi: 10.1007/s40820-023-01077-7 (PMC10102278; doi:10.1007/s40820-023-01077-7)
Supplement: Supplementary file 1 — Supplementary file1 (PDF 2270 KB) [file 40820_2023_1077_MOESM1_ESM.pdf]

Supporting Information for

Ion-Electron Coupling Enables Ionic Thermoelectric Material with New Operation Mode and High Energy Density

Yongjie He[1], Shaowei Li[1], Rui Chen[1], Xu Liu[1], George Omololu Odunmbaku[1], Wei Fang[1], Xiaoxue Lin[1], Zeping Ou[1], Qianzhi Gou[1], Jiacheng Wang[1], Nabonswende Aida Nadege Ouedraogo[1], Jing Li[1], Meng Li[1], Chen Li[1], Yujie Zheng[1], Shanshan Chen[1], Yongli Zhou[1], Kuan Sun[1,]*

[1] MOE Key Laboratory of Low-grade Energy Utilization Technologies and Systems, CQU-NUS Renewable Energy Materials & Devices Joint Laboratory, School of Energy & Power Engineering, Chongqing University, Chongqing 400044, P. R. China

*Corresponding author. E-mail: kuan.sun@cqu.edu.cn (Kuan Sun)

S1 Model for Ion-electron Thermoelectric Synergistic Effect

In this section, we derive the synergistic contribution of the ionic thermodiffusion and electron drift.

The ionic flux and internal energy flux in the electrolyte system can be described by the Onsager relationship [S1, S2]:

$$J_i = L_{ii}\frac{\nabla(-\tilde{\mu}_i)}{T} + L_{iQ}\nabla\left(\frac{1}{T}\right) \quad (S1)$$

$$J_Q = \sum_i L_{Qi}\frac{\nabla(-\tilde{\mu}_i)}{T} + L_{QQ}\nabla\left(\frac{1}{T}\right) \quad (S2)$$

Where i is the ion species, J_i, $\tilde{\mu}_i$, and J_Q are the ionic flux, electrochemical potential, and the heat flux across the sample, respectively. L_{ii}, L_{iQ}, L_{Qi}, L_{QQ} are the transport coefficients and T is the temperature.

According Han et al., in an electrolyte where both anion and cation valence states are ±1, the thermopower can be expressed as [S2]:

$$S = \frac{(\hat{S}_+D_+) - (\hat{S}_-D_-)}{e(D_+ + D_-)} \quad (S3)$$

Where S is the thermopower, D is the diffusion coefficient, e is the elementary charge. $\hat{S}$ is Eastman entropy of transfer.

Assuming that both ions and electrons exist in a homogeneous conductive gel, when no potential difference exists, the distribution of ions and electrons should be uniform so that there is no electric field exists (as shown in Fig. 1a.). When a temperature difference is applied across this gel (as

shown in Fig. S1), however, ions will diffuse from the hot side to the cold side (known as the Soret effect) resulting in an ionic diffusion current (expressed as J_{Diff}) and a potential difference. At the same time, an electric field (E, purple arrow) is formed inside the conductive gel to balance the ionic diffusion and thus form a drift current (expressed as J_{Drift}). The current generated by the intrinsic Seebeck effect of the conductive gel is denoted by $J_{e\text{-}TE}$.

The general expression of ionic current density can be expressed as [S3]:

$$J_{ionic} = \sum \left[q|z_i|C_i\mu_i \frac{d\Phi}{dx} + qz_iD_i\frac{dC_i}{dx} \right] \tag{S4}$$

Where D is the diffusion coefficient, Φ is the potential, μ is the mobility, q is the charge, C is the ion concentration, and z is the ion charge.

The diffusion current can be shown as [S4-S6]:

$$J_{Diff} = \sum \left[qz_iD_i\frac{dC_i}{dx} \right] \tag{S5}$$

Re-expressing the above equation to account for anions and cations:

$$J_{Diff} = -q\left(D_+z_+\frac{dC_+}{dx} - D_-z_-\frac{dC_-}{dx} \right) \tag{S6}$$

The drift current generated within the conductive gel can be expressed as:

$$J_{Drift} = nq\mu_n \cdot E \tag{S7}$$

$$\sigma_{e-TE} = nq\mu_n \tag{S8}$$

$$J_{Drift} = \sigma_{e-TE} \cdot E = \sigma_{e-TE}\frac{dV}{dx} \tag{S9}$$

Where σ is the electrical conductivity of the gel, E is the electric field strength.

The current generated by the conductive gel due to the Seebeck effect can be expressed as:

$$J_{e-TE} = \frac{S_e\Delta T}{R} \tag{S10}$$

Under open circuit conditions, the net current is zero, then we have:

$$J = J_{e-TE} + J_{Diff} + J_{Drift} = 0 \tag{S11}$$

Generally, the current generated by the Seebeck effect of the electronic conductor is very small and the $J_{e\text{-}TE}$ current contribution can be ignored. So that Eq. (S11) can be simplified as:

$$-qA\left(D_+z_+\frac{dC_+}{dx} - D_-z_-\frac{dC_-}{dx} \right) + \sigma_{e-TE}A\frac{dV}{dx} = 0 \tag{S12}$$

Then we have:

$$\frac{dV}{dx}=\frac{q}{\sigma_{e-TE}}\left(D_+z_+\frac{dC_+}{dx}-D_-z_-\frac{dC_-}{dx}\right) \tag{S13}$$

When we define macroscopically, it can be written as:

$$\frac{\Delta V}{\Delta x}=\frac{q}{\sigma_{e-TE}}\left(D_+z_+\frac{\Delta C_+}{\Delta x}-D_-z_-\frac{\Delta C_-}{\Delta x}\right) \tag{S14}$$

And then,

$$\Delta V=\frac{q}{\sigma_{e-TE}}(D_+z_+\Delta C_+-D_-z_-\Delta C_-) \tag{S15}$$

According to the Soret effect of ion diffusion under a temperature difference, the current density generated by the mobile ions can be written as [S7]:

$$J=-D\Delta C-D_TC\Delta T \tag{S16}$$

Where D_T is thermodiffusion coefficient.

Under equilibrium conditions [S7]:

$$\Delta C=-\,CS_T\Delta T \tag{S17}$$

Where $S_T=D_T/\mathrm{D}$ is the Soret coefficient.

In the case of equal charge numbers of anions and cations:

$$\Delta V=\frac{q}{\sigma_{e-TE}}(D_-C_-S_{T-}\Delta T\ -\ D_+C_+S_{T+}\Delta T) \tag{S18}$$

Therefore, we can obtain an expression for the Seebeck coefficient under the cooperative work of ion -electrons:

$$S=\frac{\Delta V}{\Delta T}=\frac{q}{\sigma_{e-TE}}(D_-C_-S_{T-}\ -\ D_+C_+S_{T+}) \tag{S19}$$

Note that:

$$\Delta V\propto(D_-C_-S_{T-}-D_+C_+S_{T+}) \tag{S20}$$

$$\Delta V\propto\frac{1}{\sigma_{e-TE}} \tag{S21}$$

We can conclude that the voltage under ion-electron cooperation is positively correlated with the Soret effect (Eq. S20) and negatively correlated with the conductivity of the gel (Eq. S21).

S2 Supplementary Figures and Tables

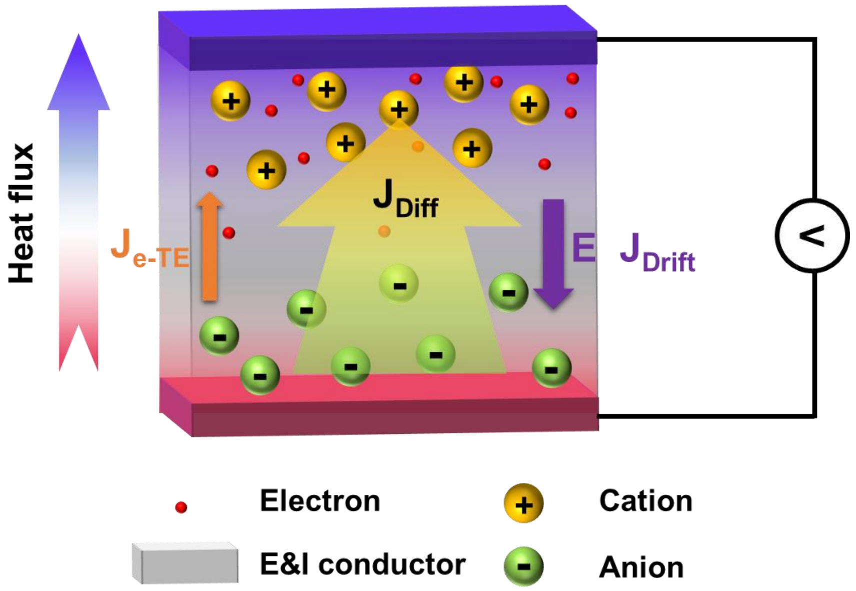

Fig. S1 Model analysis of ion-electron thermoelectric system. Schematic diagram of particle motion in an ion-electron conductor after applying a temperature difference

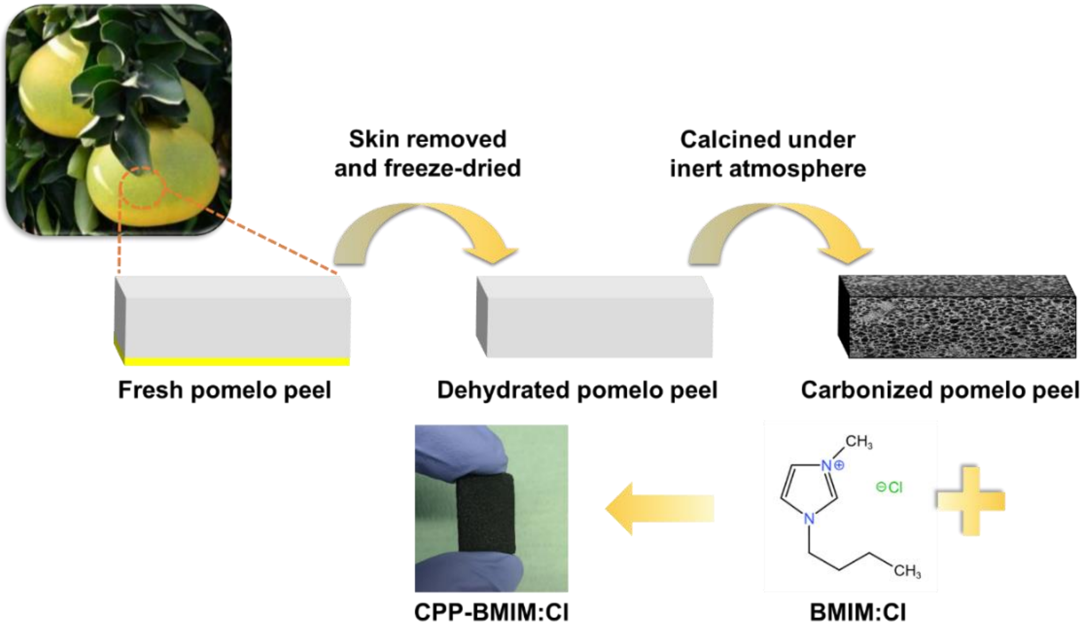

Fig. S2 Schematic illustration of the preparation process of CPP-BMIM:Cl

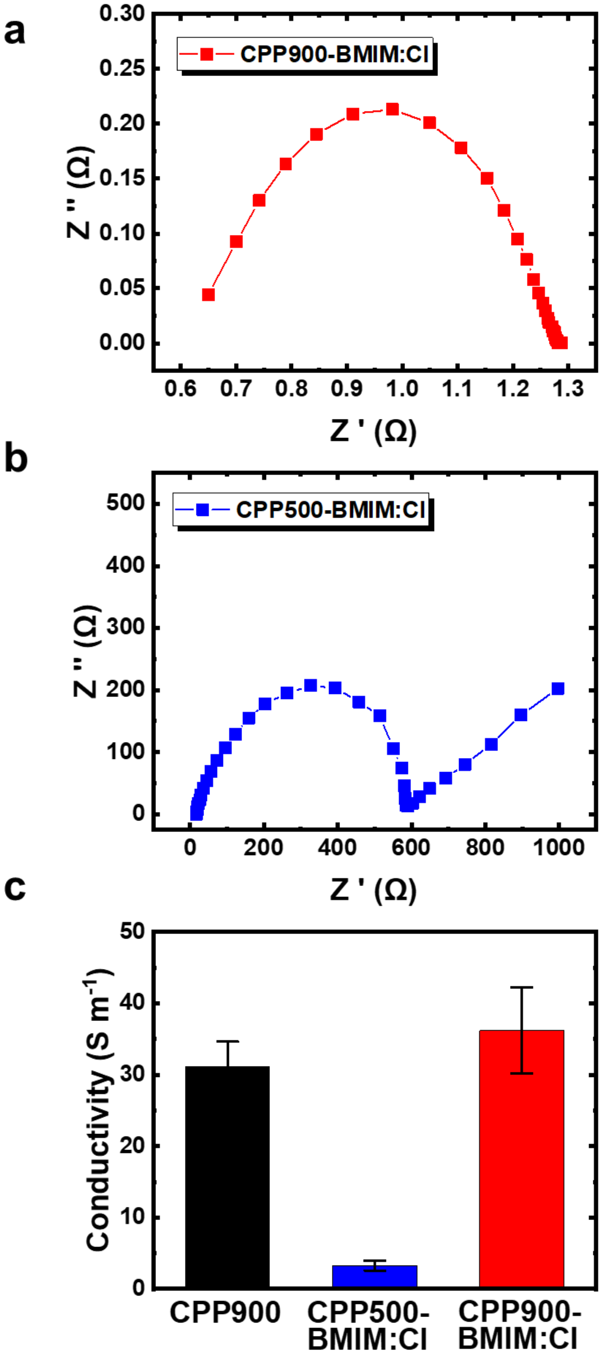

Fig. S3 Nyquist plots of **a,** the CPP900-BMIM:Cl and **b,** CPP500-BMIM:Cl. **c** Comparison of conductivity between CPP900, CPP900-BMIM:Cl and CPP500-BMIM:Cl. It shows that the conductivity of the composite is improved when BMIM:Cl is introduced

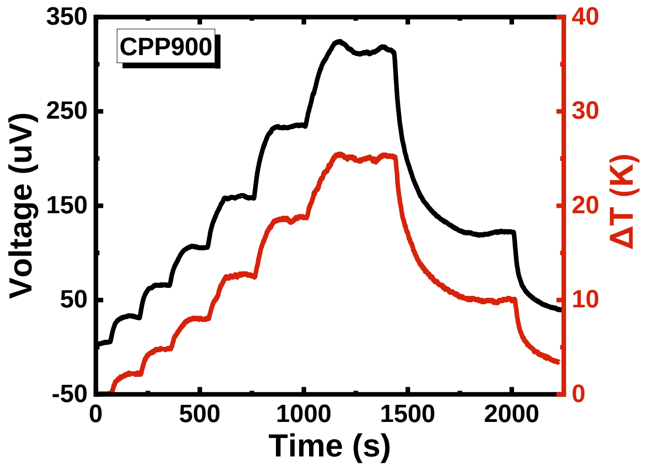

Fig. S4 The output voltage curve of CPP900 under a thermal gradient ramp; the thermopower is obtained by dividing the potential difference by the temperature difference. The results show that the thermopower of CPP900 is 12.5 μV K^{-1}, which is a typical performance of e-TE

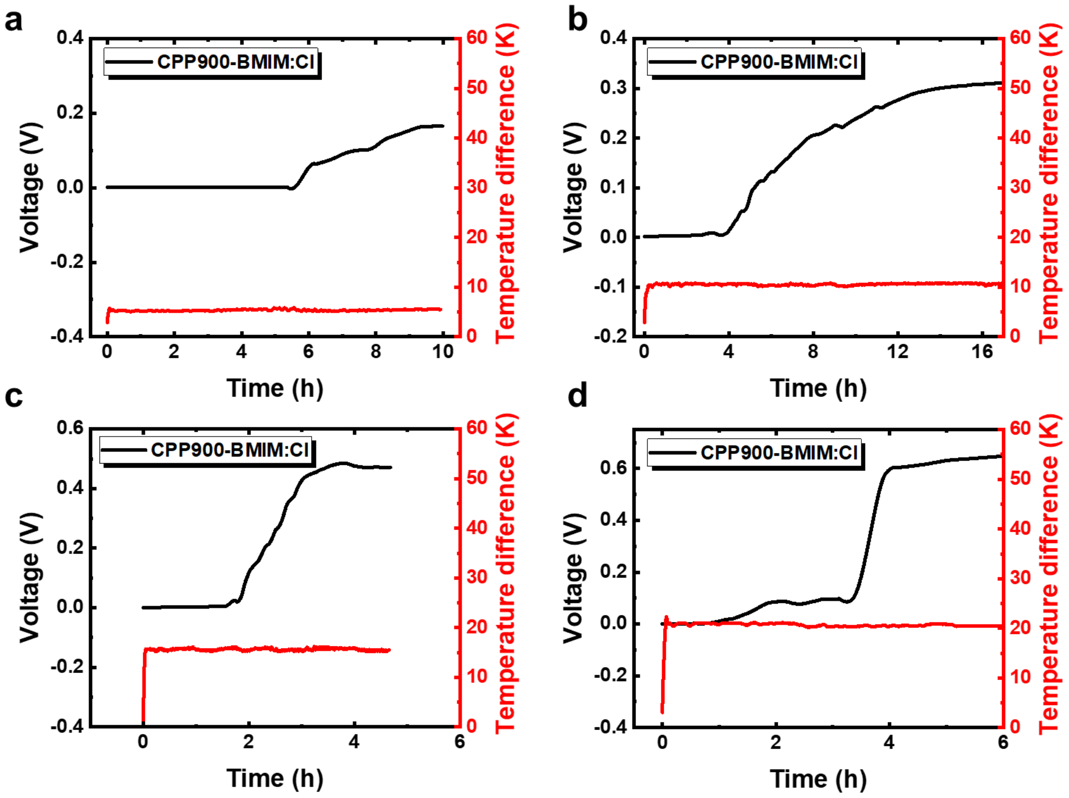

Fig. S5 Voltage-time curve of CPP900-BMIM:Cl under different constant temperature differences. When the temperature difference is 5 K, 10 K, 15 K, and 20 K, the thermal voltage reaches **a** 168.9 mV, **b** 313.3 mV, **c** 492.1 mV and **d** 655.6 mV, respectively. Thus, the thermopower of CPP900-BMIM:Cl obtained by linear fitting is 32.7 mV K^{-1}

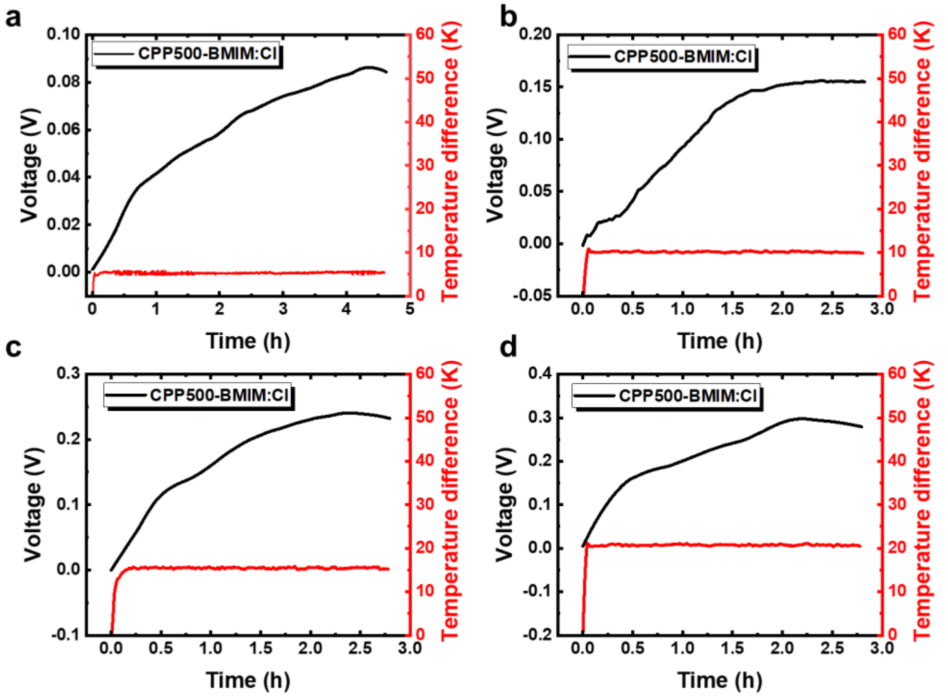

Fig. S6 Voltage-time curve of CPP500-BMIM:Cl under different constant temperature differences. When the temperature difference is 5 K, 10 K, 15 K, and 20 K, the thermal voltage reaches **a** 82.8 mV, **b** 158.4 mV, **c** 251 mV and **d** 312 mV, respectively. Thus, the thermopower of CPP500-BMIM:Cl obtained by linear fitting is 15.5 mV K^{-1}

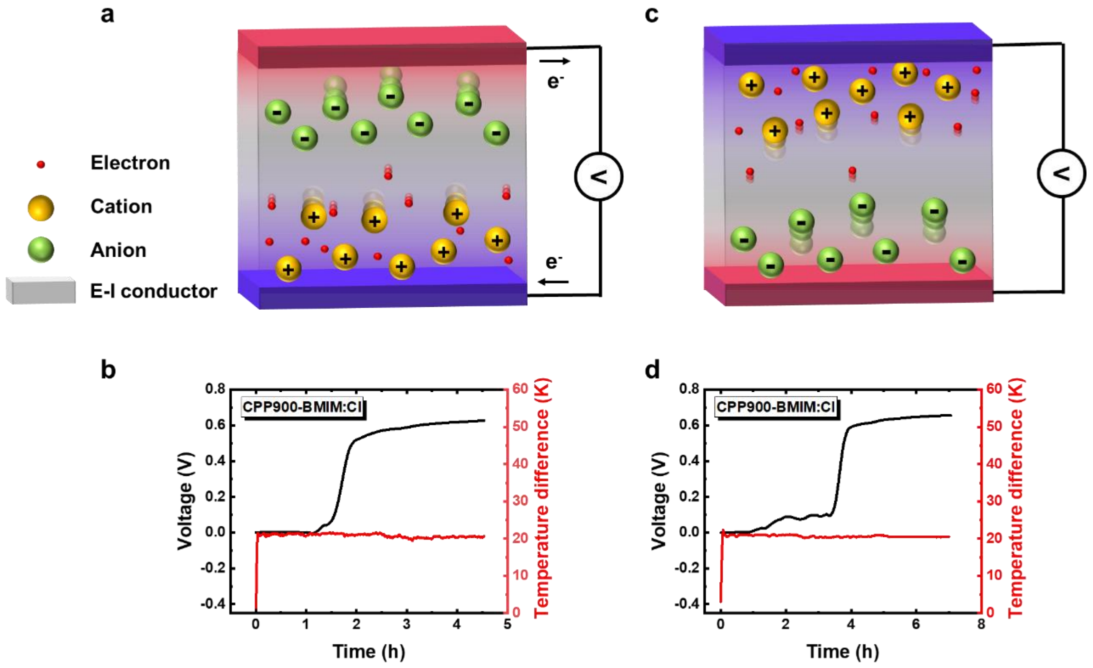

Fig. S7 The thermovoltage response after exchanging the hot and cold sides, indicating that the generation of thermovoltage is related to the direction of the temperature difference. **a** Schematic

diagram and **b** result of the thermopower test, with the cold side swapped below and the hot side on top. **c** Schematic diagram and **d** result of the thermopower test, with the hot side swapped below and the cold side on top. The test results after changing the direction of the temperature difference are equivalent to the original thermopower. Note that the temperature difference is +20 K, and the voltage is also positive, which proves the consistency between the temperature difference and the thermovoltage.

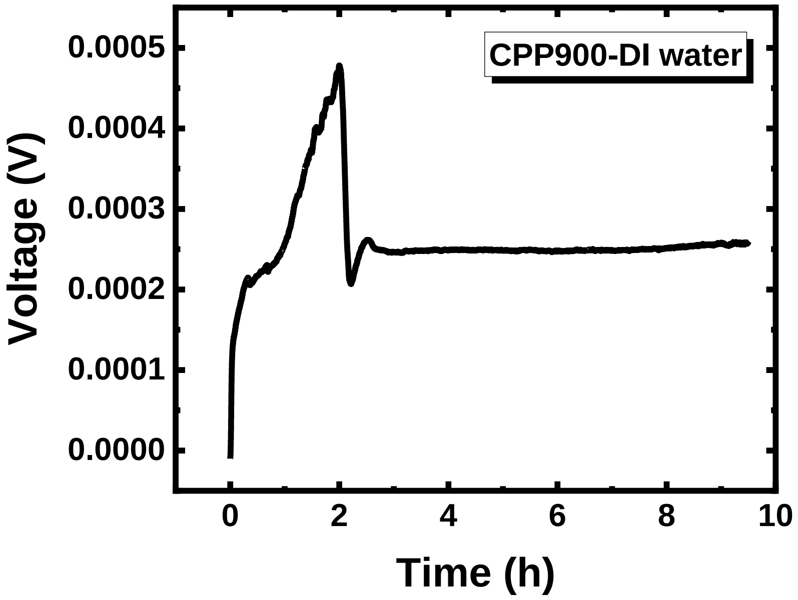

Fig. S8 The voltage test curve at a temperature difference of 20 K when only DI water is introduced, indicating that voltage generation is not dependent on water content and/or evaporation. Because previous studies have shown that in some porous carbon structures, the evaporation of water may also cause a potential difference, so it is necessary to exclude the possibility of the potential difference caused by the evaporation of water [S4, S8]. The initial rise in voltage may be caused by the evaporation of water or the movement of a small number of ions (such as H^+ on the carboxyl group) inside the CPP. As the moisture is depleted, the voltage eventually plateaus to a plateau value, which is the intrinsic thermoelectric effect of the CPP900

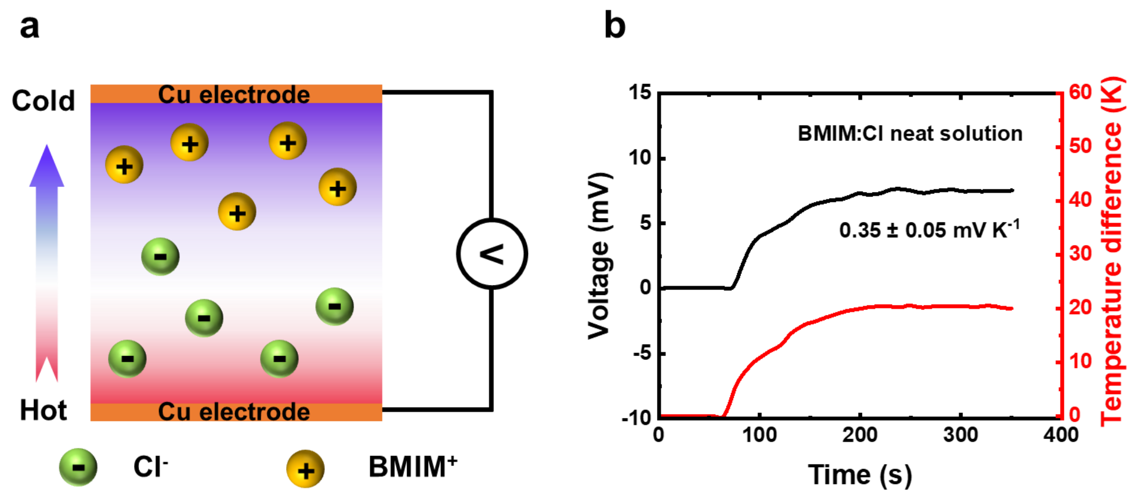

Fig. S9 The thermopower of BMIM:Cl (1 M) neat solution. The concentration of BMIM:Cl solution, electrode and ambient temperature used in the test were all consistent with the CPP-BMIM test, but the difference was whether CPP was present or not. **a** Schematic of thermopower test of BMIM:Cl solution. **b** Voltage & temperature difference-time curve of BMIM:Cl. The thermopower of 1 M BMIM:Cl neat solution is about 0.35 ± 0.05 mV K^{-1}. The obvious difference in thermopower between CPP-BMIM:Cl and BMIM:Cl solution proves that CPP plays a key role

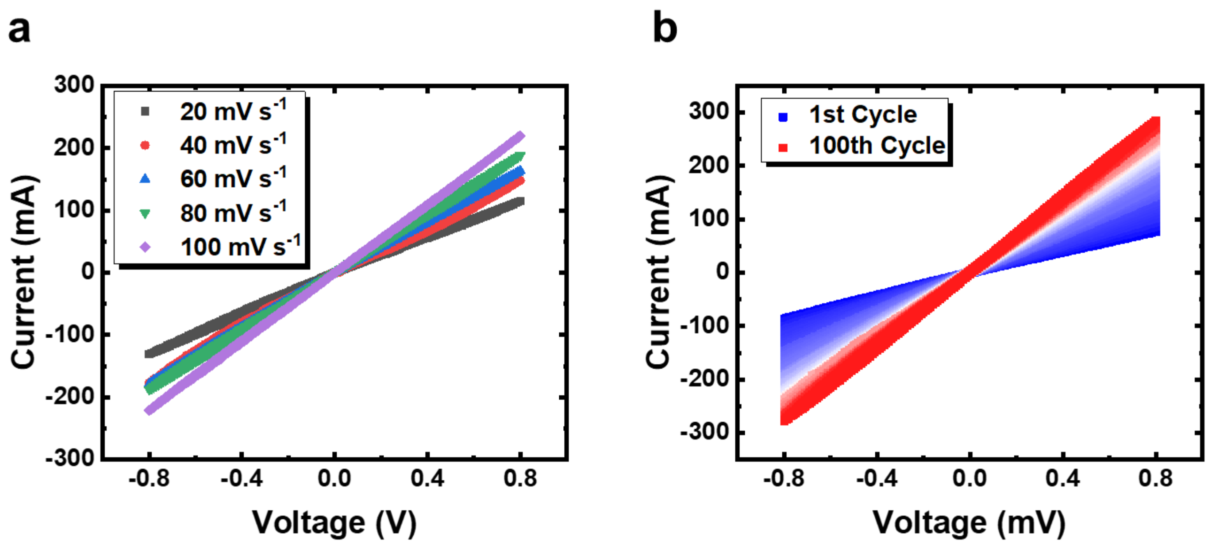

Fig. S10 **a** Cyclic voltammograms of CPP900-BMIM:Cl at different scan rates. **b** CV test of CPP900-BMIM:Cl cycled 100 times at a scan rate of 100 mV s^{-1}. No obvious redox peaks observed, suggesting that there is no redox reaction occurring within the cell. The maximum current density of Fig. S10a-b is 736 and 916 A m^{-2}, respectively. Note that the current density here is generated by the electrochemical workstation. The reason that the current is so high is probably because the CPP900 itself has very low resistance and high conductivity (31.2 S m^{-1})

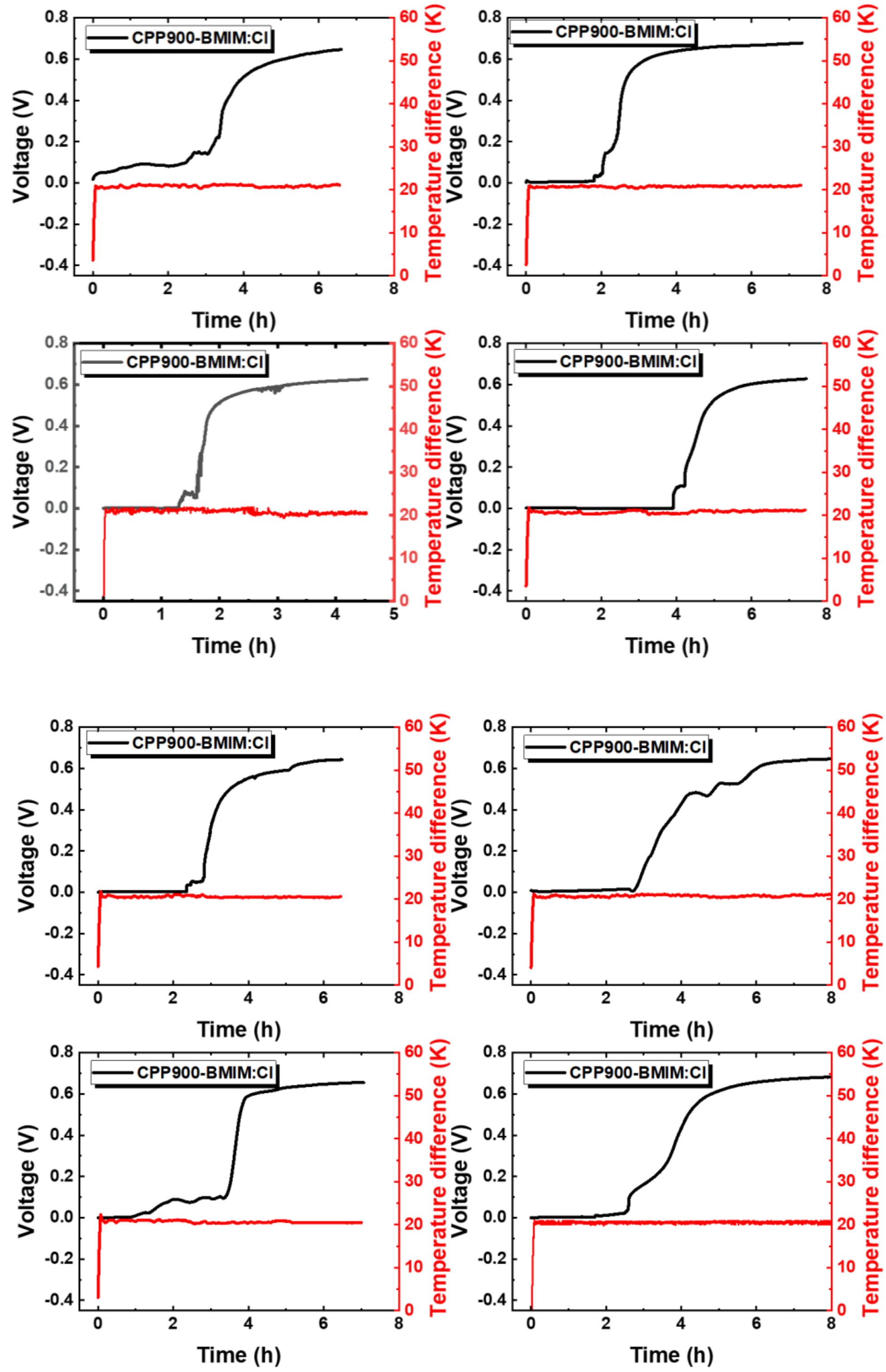

Fig. S11 Thermovoltage response of various CPP900-BMIM:Cl samples all exhibiting an initial time-lag preceding a buildup in thermovoltage (Tested in the same conditions)

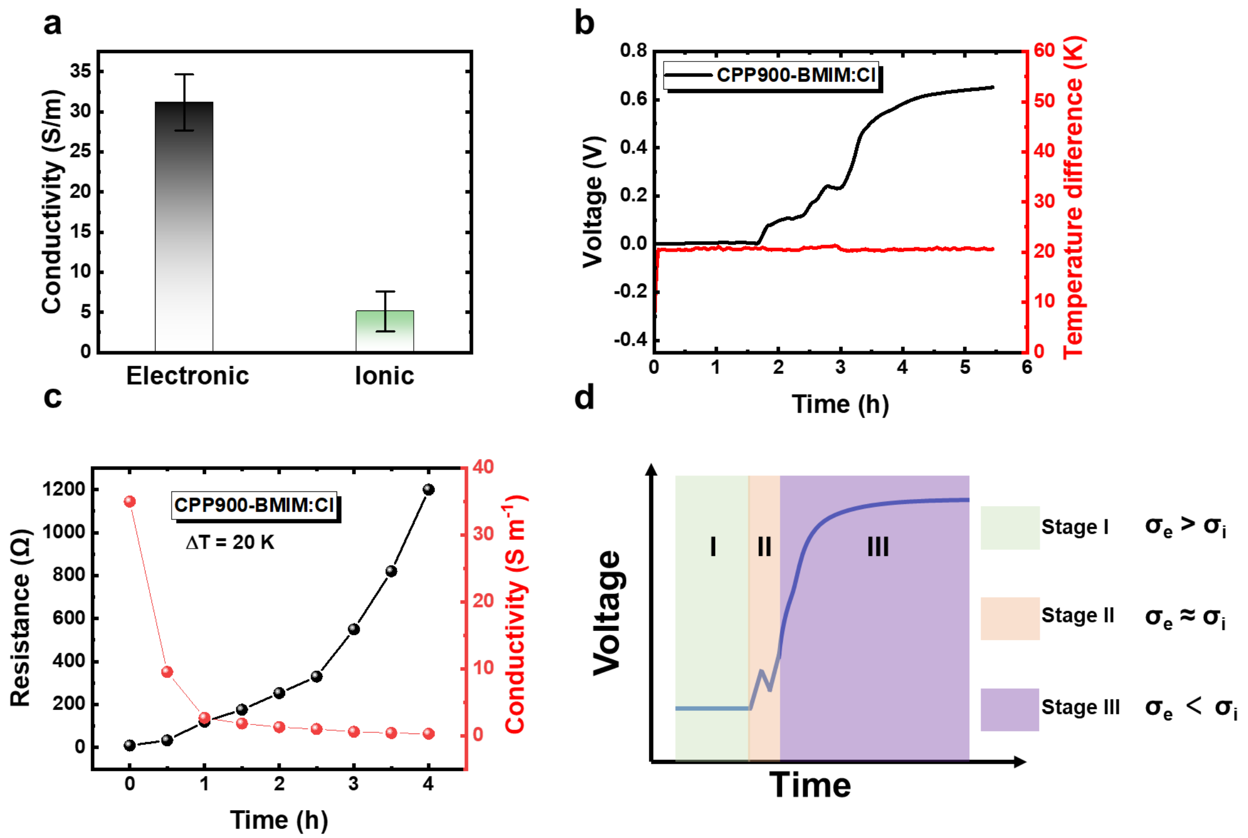

Fig. S12 The rising trend of thermal voltage of CPP900-BMIM:Cl. **a** In the beginning, the electrical conductivity is much higher than the ionic conductivity. **b** The rising trend of thermal voltage under temperature difference of 20 K. **c** The rise of resistance and the decrease in conductivity during thermal diffusion of ions. **d** The 3 stages of the thermoelectric potential of CPP900-BMIM:Cl

The thermal voltage of CPP900-BMIM:Cl apparently lags behind the temperature difference (Fig. S11) because the electrical conductivity has a decreasing process during the test. The conductivity of electronic conductors is initially higher than the ionic conductivity (Fig. S12a). This means that the thermoelectric potential generated by the ions can be quickly balanced out by the electrons in the drift electric field. Therefore, for a short period of time after the temperature difference is applied, the potential difference is only a few millivolts (Fig. S12b). With thermal diffusion of ions and dissipation of electrons in the drift electric field, the resistance of the entire device increases (Fig. S12c), exhibiting a decrease in electrical conductivity. A drop in electrical conductivity can lead to a rise in thermovoltage, which can be explained by our theoretical IETS models. Firstly, since the net current is 0, the magnitude of the drift and diffusion currents are equal as seen in the expression below:

$$J = J_{Diff} + J_{Drift} = 0 \tag{S22}$$

$$J_{Diff} = -J_{Drift} \tag{S23}$$

Then according to the expression for the drift current:

$$J_{Drift} = \sigma_{e-TE} \cdot E = \sigma_{e-TE}\frac{dV}{dx} \tag{S24}$$

Therefore, when the electrical conductivity (σ_{e-TE}) decreases, the thermovoltage (dV) rises in the case where the ions continue to diffuse.

When the electrical conductivity drops to be comparable with the ionic conductivity, there is an unstable increase in voltage. When the conductivity of the electronic conductor decreases further, the ability of electrons to balance the thermoelectric potential of the ions gradually weakens, resulting in a large increase in the thermoelectric potential of the ions. The thermopower of the electron/ion hybrid system is determined by the charge carrier with higher conductivity, this is consistent with the previous study by Crispin et al [S9].

Therefore, we can reasonably model the change in electrical conductivity and the rise in thermovoltage. Thus, thermovoltage rise can be divided into three stages (Fig. S12d). In stage I, the electrical conductivity is higher than the ionic conductivity. Electrons quickly balance the potential difference created by the ions, and the voltage is expressed as a few millivolts. In stage II, the electrical conductivity gradually drops to be comparable to the ionic conductivity. The ionic thermoelectric potential begins to grow erratically. In stage III, the electrical conductivity drops further. The ability of electrons to balance the potential difference of the ions is minimized, and the electrons cannot quickly balance the potential difference generated by the ions, which is manifested as a rise in voltage

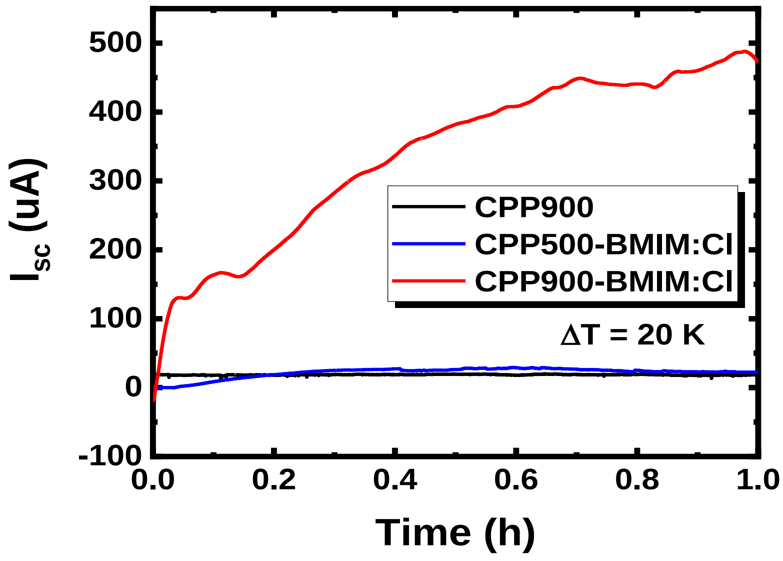

Fig. S13 Short-circuit current curve of CPP-based samples after being subjected to a temperature difference of 20 K, indicating that energy conversion occurs when there is a temperature difference

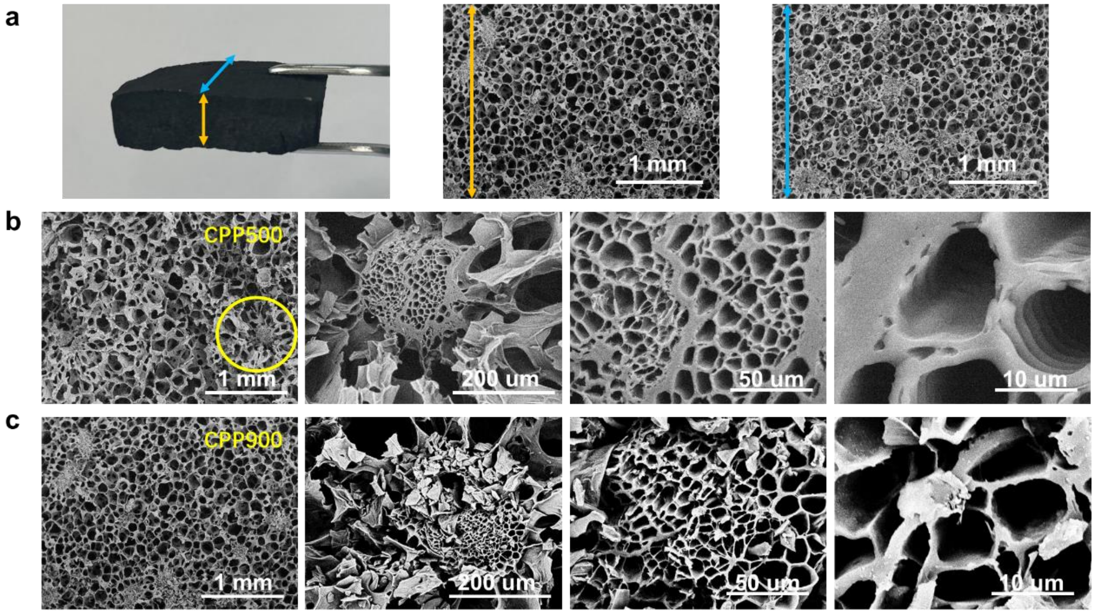

Fig. S14 a Isotropic Micro-Nano Structure of CPP. The yellow arrows represent the radial direction of the pomelo peel. The blue arrow represents the axial direction of the growth of the pomelo peel. SEM images of **b,** CPP500 and **c,** CPP900. A comparison between CPP500 and CPP900 shows that CPP900 possesses thinner and higher density nanofibers

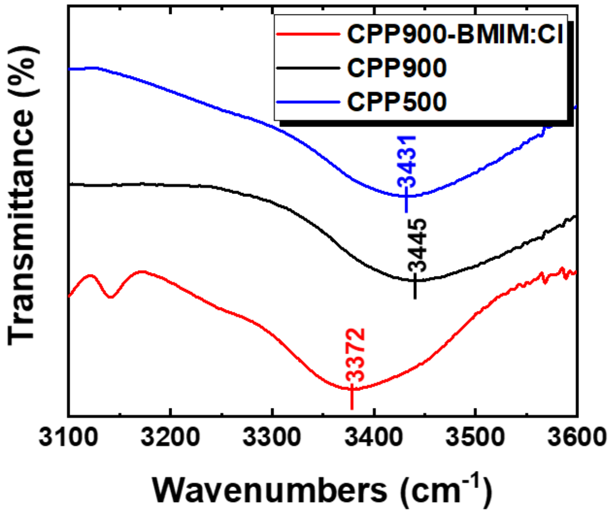

Fig. S15 FTIR spectra of CPP900, CPP900-BMIM:Cl and CPP500-BMIM:Cl in the wavenumber region between 3100 and 3600 cm^{-1}

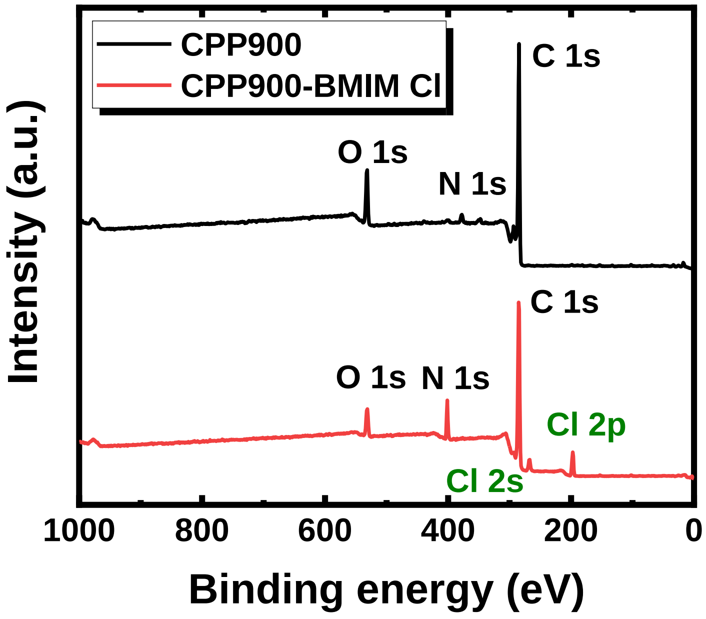

Fig. S16 XPS spectra of CPP900 and CPP900-BMIM:Cl

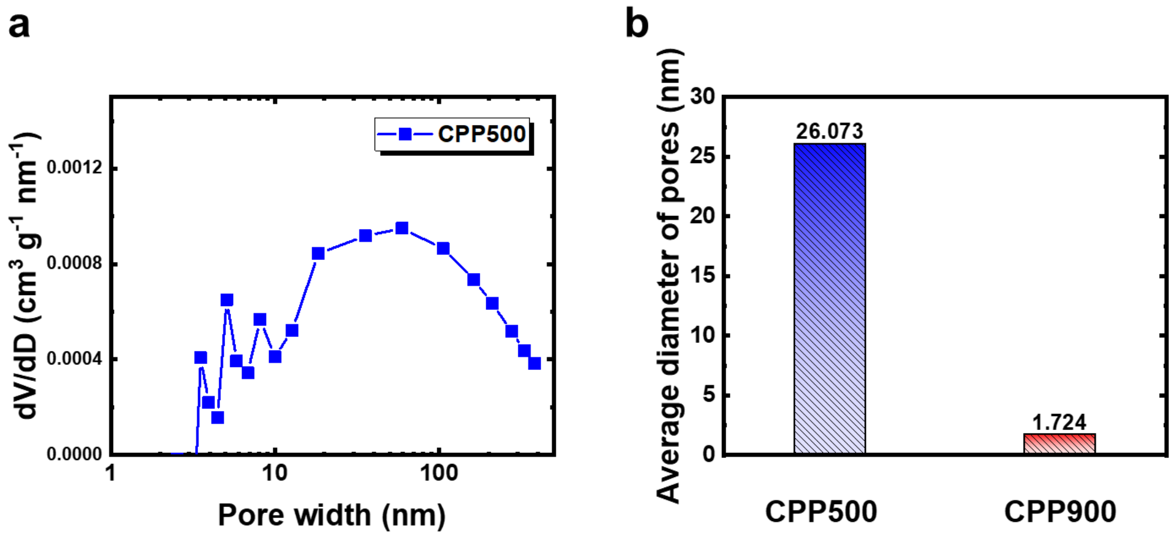

Fig. S17 a Pore size distribution of the CPP500. **b** Average nano fiber pore diameter of CPP900 and CPP500

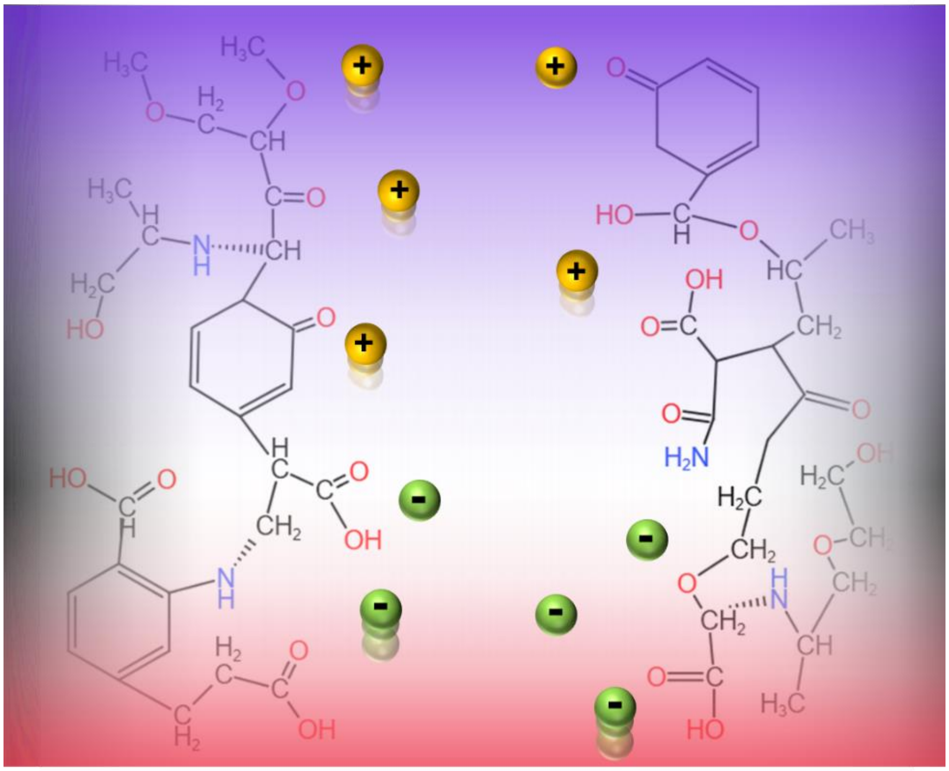

Fig. S18 Schematic figure of the diffusion and interaction of ions in CPP

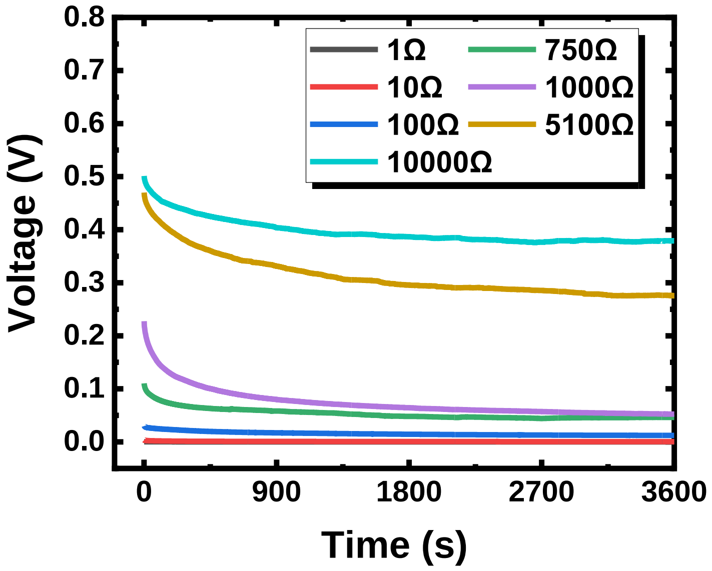

Fig. S19 The voltage curves of CPP900-BMIM:Cl during discharging when connected to different external loads

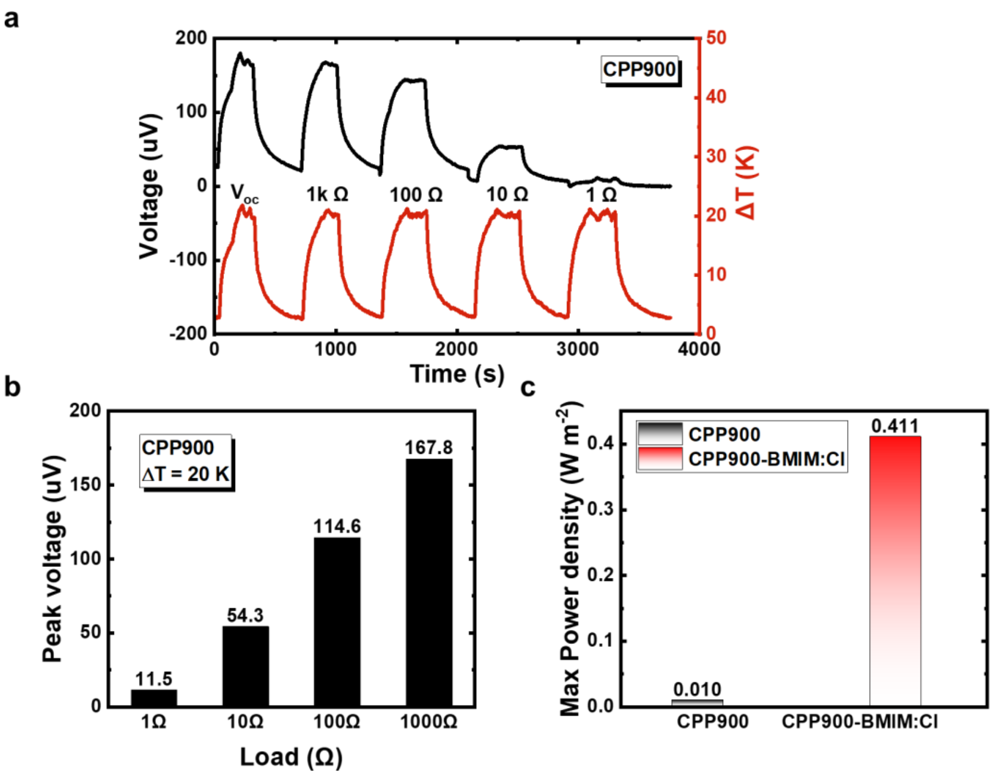

Fig. S20 Output characteristics of the CPP900. **a** Voltage curve of CPP900 with external load under the temperature difference of 20 K. **b** External load and output peak voltage of CPP900. It can be seen that the voltage and power output by the CPP900 are relatively low. **c** Maximum power density of CPP900 and CPP900-BMIM:Cl

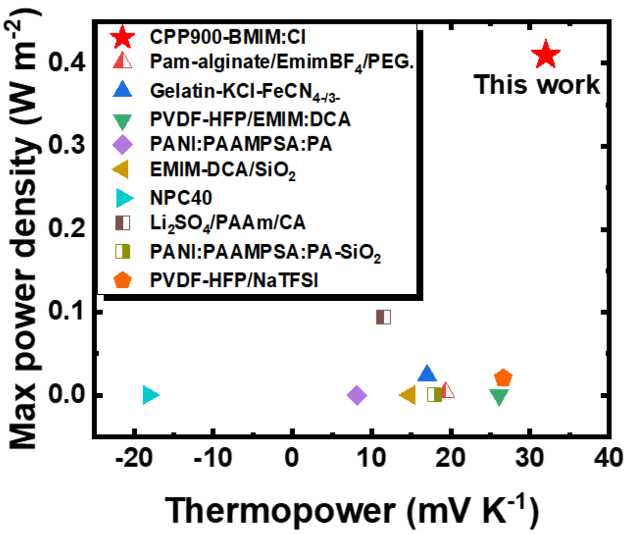

Fig. S21 Comparison of maximum power density of CPP900-BMIM:Cl with other i-TE materials reported in literature

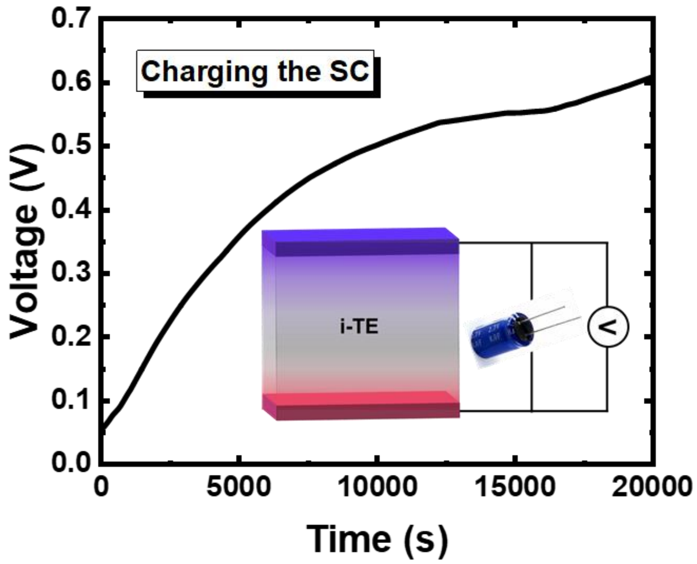

Fig. S22 The charging profile of CPP900-BMIM:Cl connected with a supercapacitor (SC) at a temperature difference of 20 K. The capacitance of the SC is 0.5 F

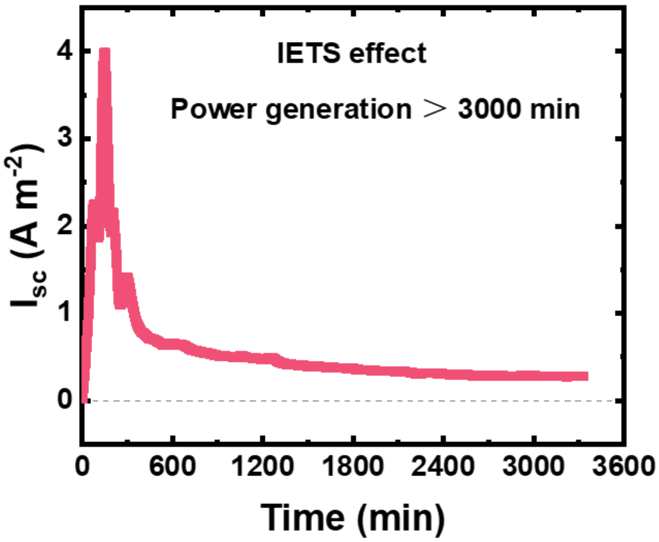

Fig. S23 The short-circuit current of CPP900-BMIM:Cl under a continuous temperature difference of 20 K

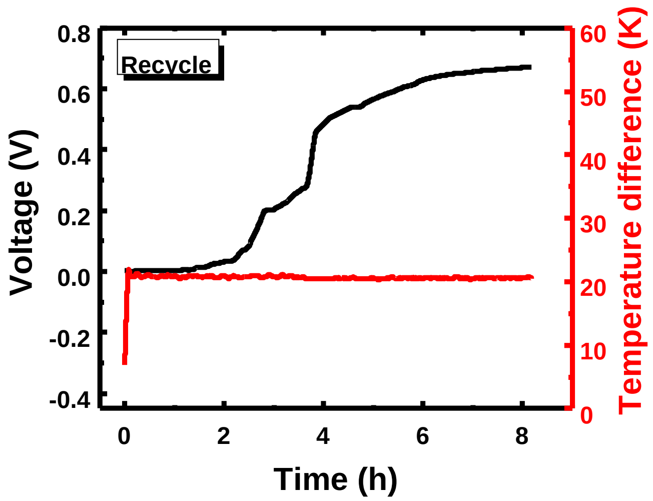

Fig. S24 Thermovoltage of restored CPP900-BMIM:Cl. After 3000 minutes of continuous power generation, the sample was immersed in an aqueous solution of 1 M BMIM:Cl for 15 min, then normal operation can be restarted and the thermovoltage can reach a peak value of 0.65 V again

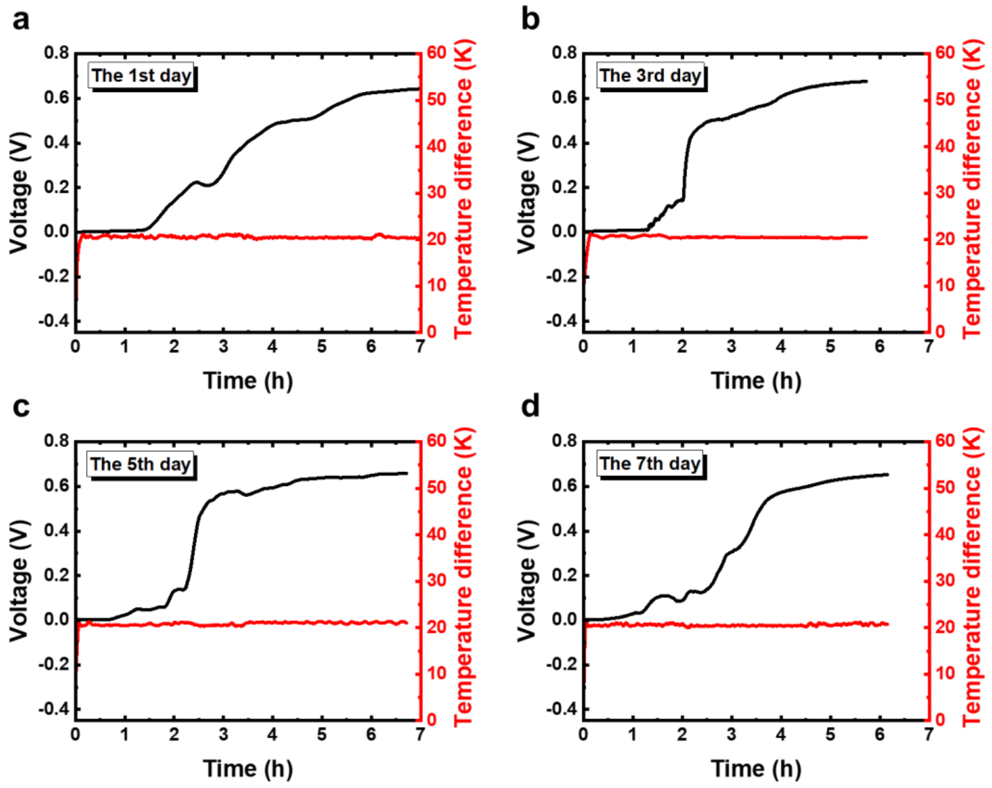

Fig. S25 Thermovoltage obtained of CPP900-BMIM:Cl over several days. **a** The 1st day, **b** 3rd day, **c** 5th day and **d** 7th day. The sample was stored in an aqueous solution of 1 M BMIM:Cl. A week-long test proves that the sample can be reused for a long time. Therefore, it is not a one-time energy source

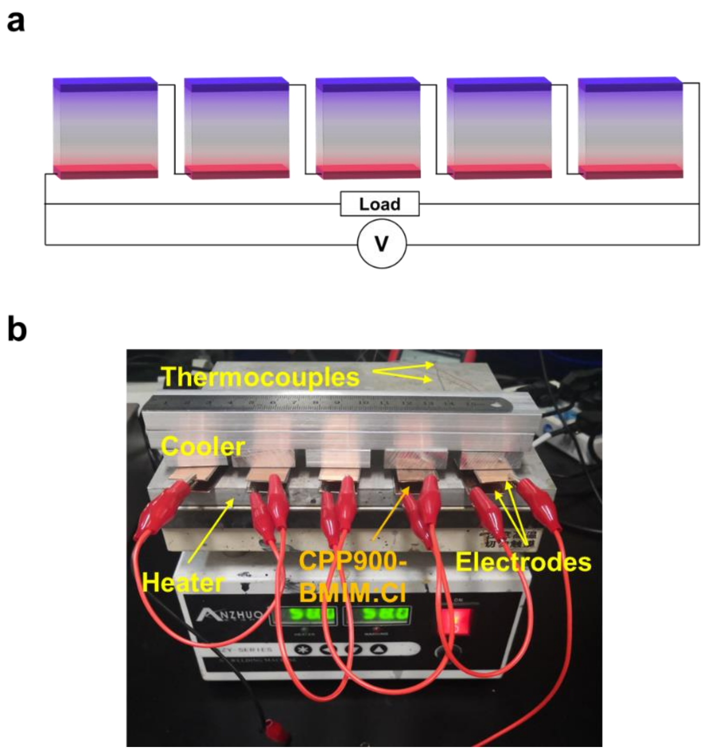

Fig. S26 a Circuit diagram of a thermoelectric module consisting of 5 units (size ~5 mm × 9 mm × 14 mm) connected in series. **b** Photograph of a CPP900-BMIM:Cl module containing 5 units in series

Table S1 Absolute thermovoltage of a single thermoelectric leg compared to literature

Carrier type	TE materials	Temperature difference (K)	|Vmax| (mV)	Refs.
Ions/electrons	CPP/BMIM:Cl	20	655	This work
Ions	Cellulose/PEO/NaOH	15	360	[S10]
Ions	PVDF-HFP/EMIM:DCA	0.5	14	[S11]
Ions	PEO/NaOH	5	58	[S12]
Ions	Gelatin/KCl& $FeCN^{4-/3-}$	9.5	120	[S2]
Ions	PVDF-HFP/PEG/EMIM:TFSI	6	84	[S13]
Ions	GdmCl/$FeCN^{4-/3-}$	50	186.5	[S14]
Ions	Urea/GdmCl/$FeCN^{4-/3-}$	30	126	[S15]
Ions/electrons	Perylene bisimide (PBI)	10	63	[S16]
Electrode corrosion	PANI:PSS	5.2	480	[S17]

Table S2 Comparison of energy density and power density of CPP900-BMIM:Cl with literature

Material	Thermopower (mV K^{-1})	Temperature difference (K)	Energy density (J m^{-2})	Power density (W m^{-2})	Refs.
CPP900-BMIM:Cl	32	20	553.9	0.41	This work
PEO/NaOH	11	4.5	1.35×10^{-2}		[S12]
Gelatin-KCl-$FeCN^{4-/3-}$	17	8	12.8	About 0.024	[S2]
Pam-alginate/EmimBF4/PEG.	19.32			0.0031	[S18]
PVDF-HFP/EMIM:DCA	26.1	0.6	2.24×10^{-4}	0.84×10^{-6}	[S19]
PANI:PAAMPSA:PA	8.1	1.7		2.38×10^{-6}	[S20]
EMIM-DCA/SiO_2	14.8	0.5	0.25	1.8×10^{-4}	[S21]
NPC40	-18.2	4.5		3.8×10^{-4}	[S22]
Li_2SO_4/PAAm/CA	11.5	40		9.4×10^{-2}	[S23]
PSSH/GO	13	9.5	2		[S24]
PANI:PAAMPSA:PA-SiO_2	17.9	3	2.71×10^{-3}	4.4×10^{-5}	[S25]
PVDF-HFP/NaTFSI	26.62	1.5	8.33×10^{-2}	2.08×10^{-2}	[S26]
Gelatin-KCl-$FeCN^{4-/3-}$ (3D Au/Cu)	17	9	80		[S27]

Supplementary Video S1 i-TE cells drive thermo-hygrometer continuously at $\Delta T = 20$ K

Supplementary References

[S1] M. Bonetti, S. Nakamae, M. Roger, P. Guenoun. Huge seebeck coefficients in nonaqueous electrolytes. J. Chem. Phys. **134**(11), 114513 (2011). https://doi.org/10.1063/1.3561735

[S2] C.-G. Han, X. Qian, Q. Li, B. Deng, Y. Zhu, Z. Han, W. Zhang, W. Wang, S.-P. Feng, G. Chen, W. Liu. Giant thermopower of ionic gelatin near room temperature. Science **368**(6495), 1091-1097 (2020). https://doi.org/10.1126/science.aaz5045

[S3] B. D. Paulsen, S. Fabiano, J. Rivnay. Mixed ionic-electronic transport in polymers. Annu Rev. Mater. Sci. **51**(1), 73-99 (2021). https://doi.org/10.1146/annurev-matsci-080619-

101319

[S4] H. Cheng, Y. Huang, F. Zhao, C. Yang, P. Zhang et al., Spontaneous power source in ambient air of a well-directionally reduced graphene oxide bulk. Energy Environ. Sci. **11**(10), 2839-2845 (2018). https://doi.org/10.1039/c8ee01502c

[S5] X. Liu, H. Gao, J. E. Ward, X. Liu, B. Yin et al., Power generation from ambient humidity using protein nanowires. Nature **578**(7796), 550-554 (2020). https://doi.org/10.1038/s41586-020-2010-9

[S6] Q. Liu, Y. Wang, W. Guo, H. Ji, J. Xue et al., Asymmetric properties of ion transport in a charged conical nanopore. Phys. Rev. E Stat. Nonlin. Soft Matter Phys. **75**(5 Pt 1), 051201 (2007). https://doi.org/10.1103/PhysRevE.75.051201

[S7] M. Massetti, F. Jiao, A. J. Ferguson, D. Zhao, K. Wijeratne et al., Unconventional thermoelectric materials for energy harvesting and sensing applications. Chem. Rev. **121**(20), 12465-12547 (2021). https://doi.org/10.1021/acs.chemrev.1c00218

[S8] G. Xue, Y. Xu, T. Ding, J. Li, J. Yin et al., Water-evaporation-induced electricity with nanostructured carbon materials. Nat. Nanotechnol. **12**(4), 317-321 (2017). https://doi.org/10.1038/nnano.2016.300

[S9] H. Wang, U. Ail, R. Gabrielsson, M. Berggren, X. Crispin. Ionic seebeck effect in conducting polymers. Adv Energy Mater. **5**(11), 1500044 (2015). https://doi.org/10.1002/aenm.201500044

[S10] T. Li, X. Zhang, S. D. Lacey, R. Mi, X. Zhao et al., Cellulose ionic conductors with high differential thermal voltage for low-grade heat harvesting. Nat. Mater. **18**(6), 608-613 (2019). https://doi.org/10.1038/s41563-019-0315-6

[S11] H. Cheng, X. He, Z. Fan, J. Ouyang. Flexible quasi-solid state ionogels with remarkable seebeck coefficient and high thermoelectric properties. Adv. Energy Mater. **9**(32), 1901085 (2019). https://doi.org/10.1002/aenm.201901085

[S12] D. Zhao, H. Wang, Z. U. Khan, J. C. Chen, R. Gabrielsson et al., Ionic thermoelectric supercapacitors. Energy Environ. Sci. **9**(4), 1450-1457 (2016). https://doi.org/10.1039/c6ee00121a

[S13] D. Zhao, A. Martinelli, A. Willfahrt, T. Fischer, D. Bernin et al., Polymer gels with tunable ionic seebeck coefficient for ultra-sensitive printed thermopiles. Nat. Commun. **10**(1), 1093 (2019). https://doi.org/10.1038/s41467-019-08930-7

[S14] B. Yu, J. Duan, H. Cong, W. Xie, R. Liu et al., Thermosensitive crystallization-boosted liquid thermocells for low-grade heat harvesting. Science **370**(6514), 342-346 (2020). https://doi.org/10.1126/science.abd6749

[S15] J. Duan, G. Feng, B. Yu, J. Li, M. Chen et al., Aqueous thermogalvanic cells with a high seebeck coefficient for low-grade heat harvest. Nat. Commun. **9**(1), 5146 (2018). https://doi.org/10.1038/s41467-018-07625-9

[S16] Q. Jiang, H. Sun, D. Zhao, F. Zhang, D. Hu et al., High thermoelectric performance in n-type perylene bisimide induced by the soret effect. Adv. Mater. **32**(45), 2002752 (2020). https://doi.org/10.1002/adma.202002752

[S17] Y. Zhang, A. Sohn, A. Chakraborty, C. Yu. Colossal thermo-hydro-electrochemical voltage generation for self-sustainable operation of electronics. Nat. Commun. **12**(1), 5269 (2021). https://doi.org/10.1038/s41467-021-25606-3

[S18] C. Liu, Q. Li, S. Wang, W. Liu, N. X. Fang et al., Ion regulation in double-network hydrogel module with ultrahigh thermopower for low-grade heat harvesting. Nano Energy **92**, 106738 (2022). https://doi.org/10.1016/j.nanoen.2021.106738

[S19] H. Cheng, X. He, Z. Fan, J. Ouyang. Flexible quasi-solid state ionogels with remarkable seebeck coefficient and high thermoelectric properties. Adv. Energy Mater. **9**(32), 1901085 (2019). https://doi.org/10.1002/aenm.201901085

[S20] Z. A. Akbar, J.-W. Jeon, S.-Y. Jang. Intrinsically self-healable, stretchable thermoelectric materials with a large ionic seebeck effect. Energy Environ Sci. **13**(9), 2915-2923 (2020). https://doi.org/10.1039/c9ee03861b

[S21] X. He, H. Cheng, S. Yue, J. Ouyang. Quasi-solid state nanoparticle/(ionic liquid) gels with significantly high ionic thermoelectric properties. J. Mater. Chem A **8**(21), 10813-10821 (2020). https://doi.org/10.1039/d0ta04100a

[S22] B. Kim, J. U. Hwang, E. Kim. Chloride transport in conductive polymer films for an n-type thermoelectric platform. Energy Environ Sci. **13**(3), 859-867 (2020). https://doi.org/10.1039/c9ee02399b

[S23] J. Chen, L. Zhang, Y. Tu, Q. Zhang, F. Peng et al., Wearable self-powered human motion sensors based on highly stretchable quasi-solid state hydrogel. Nano Energy **88**, 106272 (2021). https://doi.org/10.1016/j.nanoen.2021.106272

[S24] M. Jeong, J. Noh, M. Z. Islam, K. Kim, A. Sohn et al., Embedding aligned graphene oxides in polyelectrolytes to facilitate thermo-diffusion of protons for high ionic thermoelectric figure-of-merit. Adv. Funct. Mater. **31**, 2011016 (2021). https://doi.org/10.1002/adfm.202011016

[S25] Y. T. Malik, Z. A. Akbar, J. Y. Seo, S. Cho, S. Y. Jang et al., Self-healable organic–inorganic hybrid thermoelectric materials with excellent ionic thermoelectric properties. Adv. Energy Mater. **12**(6), 2103070 (2021). https://doi.org/10.1002/aenm.202103070

[S26] C. Chi, M. An, X. Qi, Y. Li, R. Zhang et al., Selectively tuning ionic thermopower in all-

solid-state flexible polymer composites for thermal sensing. Nat. Commun. **13**(1), 221 (2022). https://doi.org/10.1038/s41467-021-27885-2

[S27] Y. Li, Q. Li, X. Zhang, B. Deng, C. Han et al., 3d hierarchical electrodes boosting ultrahigh power output for gelatin - KCl - $FeCN^{4-/3-}$ ionic thermoelectric cells. Adv. Energy Mater. **12**, 2103666 (2022). https://doi.org/10.1002/aenm.202103666
